# Hyperthyroidism-driven bone loss depends on BMP receptor *Bmpr1a* expression in osteoblasts

**DOI:** 10.1038/s42003-024-06227-0

**Published:** 2024-05-08

**Authors:** Franziska Lademann, Eddy Rijntjes, Josef Köhrle, Elena Tsourdi, Lorenz C. Hofbauer, Martina Rauner

**Affiliations:** 1 Department of Medicine III & Center for Healthy Aging, Medical Faculty and University Hospital Carl Gustav Carus, Dresden University of Technology, Dresden, Germany; 2 Charité – Universitätsmedizin Berlin, corporate member of Freie Universität Berlin and Humboldt Universität zu Berlin, Institut für Experimentelle Endokrinologie, Berlin, Germany

**Keywords:** Thyroid diseases, Cell signalling

## Abstract

Hyperthyroidism is a well-known trigger of high bone turnover that can lead to the development of secondary osteoporosis. Previously, we have shown that blocking bone morphogenetic protein (BMP) signaling systemically with BMPR1A-Fc can prevent bone loss in hyperthyroid mice. To distinguish between bone cell type-specific effects, conditional knockout mice lacking *Bmpr1a* in either osteoclast precursors (LysM-Cre) or osteoprogenitors (Osx-Cre) were rendered hyperthyroid and their bone microarchitecture, strength and turnover were analyzed. While hyperthyroidism in osteoclast precursor-specific *Bmpr1a* knockout mice accelerated bone resorption leading to bone loss just as in wildtype mice, osteoprogenitor-specific *Bmpr1a* deletion prevented an increase of bone resorption and thus osteoporosis with hyperthyroidism. In vitro, wildtype but not *Bmpr1a*-deficient osteoblasts responded to thyroid hormone (TH) treatment with increased differentiation and activity. Furthermore, we found an elevated *Rankl*/*Opg* ratio with TH excess in osteoblasts and bone tissue from wildtype mice, but not in *Bmpr1a* knockouts. In line, expression of osteoclast marker genes increased when osteoclasts were treated with supernatants from TH-stimulated wildtype osteoblasts, in contrast to *Bmpr1a*-deficient cells. In conclusion, we identified the osteoblastic BMP receptor BMPR1A as a main driver of osteoporosis in hyperthyroid mice promoting TH-induced osteoblast activity and potentially its coupling to high osteoclastic resorption.

## Abstract (web summary)

Comparison of the bone phenotypes of hyperthyroid versus euthyroid bone cell-specific conditional knockout mice suggests that the osteoblastic, but not osteoclast, BMP receptor BMPR1A is a main driver of hyperthyroidism-associated bone disease.

## Introduction

Thyroid disorders can affect the whole body´s metabolism and energy level, heart rate, body weight, mood, and also skeletal growth and bone health^1^. Although hyperthyroidism, a condition with high circulating concentrations of thyroid hormones 3,3´,5-triiodo-L-thyronine (T_3_) and L-thyroxine (T_4_), is an established risk factor for secondary osteoporosis, the molecular mechanisms for thyroid hormone excess driven bone loss still remain incompletely understood^2^. In patients and preclinical models, hyperthyroidism leads to an increase of both, bone formation and bone resorption, overall shortening the bone turnover cycle^2,3^. Given the prevalent bone resorption and a net bone loss by 10% per cycle, hyperthyroid patients may suffer from various facets of osteoporosis such as low bone mass, impaired bone quality and higher skeletal fragility leading to an increased susceptibility to bone fractures^2,4–8^.

At the cellular level, thyroid hormones are reported to stimulate osteoblast differentiation and activity^9–15^. A few studies confirm that bone-resorbing osteoclasts express thyroid hormone receptors and take up thyroid hormones by specific transporters and thus, might respond to thyroid hormones^16–20^. Nevertheless, cytokines and paracrine factors secreted by thyroid hormone stimulated cells of the osteoblast lineage are postulated to have the greater impact on osteoclastogenesis^2,21–26^.

One of the most important osteogenic pathways that controls not only osteoblastogenesis but also osteoblast-osteoclast coupling is the bone morphogenetic protein (BMP) pathway^27,28^. BMP ligands exert their effects via serine threonine transmembrane receptors, BMP type I and type II receptors. BMP type I receptors such as BMPR1A (also ALK3) can bind BMP ligands and subsequently form a heterotetrameric receptor complex with a type 2 receptor such as BMPR2. Those receptor complexes can activate two subsequent signaling pathways: the canonical, SMAD-dependent and the non-canonical pathway involving protein kinases such p38, PI3K/AKT or MAPK^27,28^. Activated SMAD1/5/9 proteins can dimerize with SMAD4 to form transcription factor complexes that induce the expression of BMP target genes such as *Runx2* or *Id1*^27,28^.

The skeletal phenotypes of several transgenic mouse models targeting BMP ligands, BMP receptors, SMAD proteins and BMP inhibitors have been studied to decipher their role in bone growth and metabolism. As such, osteoblast- and osteocyte-specific *Bmpr1a* knockout mice present a striking osteosclerotic bone phenotype due to low bone turnover, with a drastic reduction in bone resorption exceeding the low bone formation^29–32^. This suppression of osteoclasts and thus, bone resorption is driven by a decreased ratio of receptor activator of NF-κB ligand to osteoprotegerin (RANKL/OPG), a prominent stimulator and inhibitor of osteoclastogenesis, respectively, secreted by *Bmpr1a*-deficient osteogenic cells^29^. Recent studies also confirm the importance of BMP signaling in osteoclasts, especially during maturation and in promoting their resorptive activity^33–37^.

In our previous study, we demonstrated that thyroid hormones activate SMAD-dependent BMP signaling in osteoblasts^26^. Pharmacological blockade of BMP signaling either at the receptor or ligand level impaired thyroid hormone response of osteoblasts^26^. Importantly, administration of BMPR1A-Fc, a potent BMPR1A-specific BMP ligand scavenger, prevented osteoporosis in hyperthyroid mice by normalizing both bone formation and bone resorption^26^. However, this systemic approach did not reveal which bone cell type mainly accounts for thyroid hormone actions in bone. In this study, we used male and female conditional knockout mice targeting *Bmpr1a* expression in either osteoclast precursors (LysM-Cre) or osteoprogenitors (Osx-Cre) and rendered them hyperthyroid to assess whether BMP signaling in osteoblasts or osteoclasts primarily drives the pathogenesis of hyperthyroidism-induced bone loss.

## Results

### *Bmpr1a* deletion in osteoclast precursors does not prevent hyperthyroidism-induced osteoporosis

Given the predominant bone resorption in hyperthyroid mice, we first investigated whether *Bmpr1a* in osteoclast precursors might contribute to thyroid hormone excess driven bone loss.

**Fig. 1:**
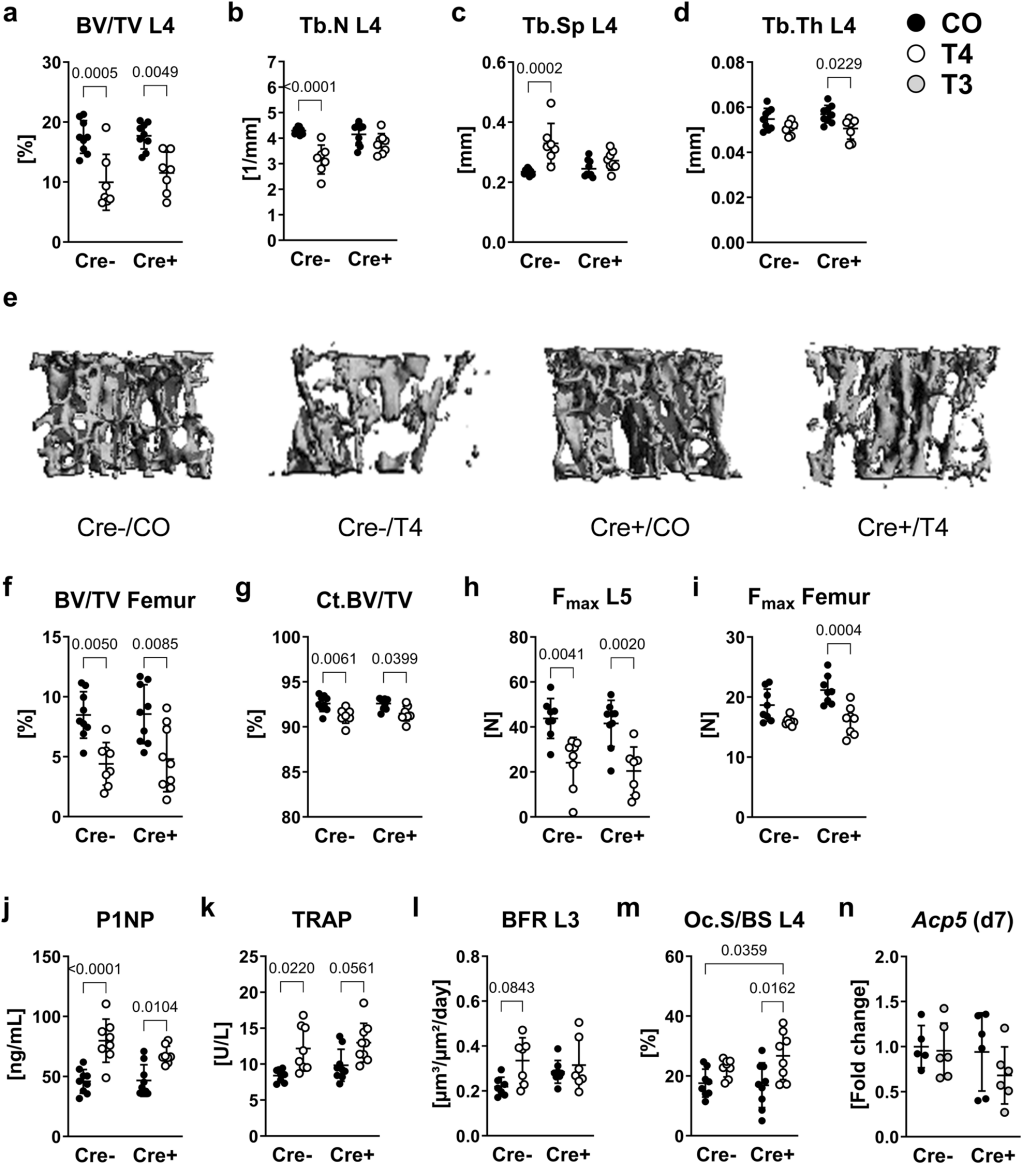
Osteoclast progenitor-specific *Bmpr1a* deletion does not prevent hyperthyroidism-induced bone loss. Twelve-week old male Cre-negative (Cre-) and Cre-positive (Cre +) *Bmpr1a*^fl/fl^;LysM-Cre mice remained euthyroid (CO) or were rendered hyperthyroid (T4) by adding 1.2 µg/mL L-thyroxine into their drinking water over 4 weeks. Using microCT analysis, (**a**) bone volume per total volume (BV/TV), (**b**) trabecular number (Tb.N), (**c**) trabecular separation (Tb.Sp), and (**d**) trabecular thickness (Tb.Th) were determined at the fourth lumbar vertebra (L4). **e** Representative 3D reconstructions of the trabecular compartment of L4. Additionally, (**f**) trabecular BV/TV and (**g**) cortical bone volume over total volume (Ct.BV/TV) were assessed at the femur. The maximal force (F_max_) as an indicator of bone strength (**h**) at the L5 vertebra and (**i**) femur was tested by compression test and 3-point bending test, respectively. Serum concentrations of (**j**) bone formation marker P1NP and (**k**) bone resorption marker TRAP were analyzed by ELISA. Furthermore, (**l**) the bone formation rate per bone surface (BFR) and (**m**) osteoclast surface per bone surface (Oc.S/BS) were assessed at the L3 and L4 vertebra, respectively. In vitro, (**n**) expression of acid phosphatase 5, tartrate resistant (*Acp5*), encoding for TRAP, in primary osteoclasts derived from *Bmpr1a*^fl/fl^;LysM-Cre mice at day 7 of differentiation, with or w/o treatment with 100 nM T_3_ (T3) over 48 h, was quantified using quantitative real-time PCR. Each dot indicates an individual mouse. In vivo: Cre-/CO: *N* = 9; Cre-/T4: *N* = 8; Cre +/CO: *N* = 9; Cre + /T4: *N* = 9; In vitro: Cre-/CO: *N* = 5; Cre-/T3: *N* = 6; Cre +/CO: *N* = 6; Cre +/T3: *N* = 6. The horizontal lines represent the mean +/- SD. Statistical analysis was performed by Two-way ANOVA and selected *p* values are shown within the graph.

Conditional *Bmpr1a* knockout in *LysM*-expressing cells led to a significant downregulation of *Bmpr1a* expression by 69.1% in untreated mature osteoclasts in vitro (Fig. S1a). Nevertheless, we did not observe any alterations of the body weight, femur length and bone phenotype in male mice due to the loss of *Bmpr1a* in osteoclast precursors (Fig. 1, Table 1). After 4 weeks of L-thyroxine treatment, serum concentrations of total T_4_ (Cre-negative: 4.1-fold; Cre-positive: 3.7-fold vs. respective untreated controls) and total T_3_ (Cre-negative: 2.2-fold; Cre-positive: 1.9-fold vs. respective untreated controls) increased in both Cre-negative and Cre-positive male *Bmpr1a*^fl/fl^;LysM-Cre mice (Fig. S2a, b). Neither body weight nor femur length were affected by hyperthyroidism (Table 1), however, we observed trabecular bone loss at the spine and femur in hyperthyroid male mice regardless of their *Bmpr1a* expression in osteoclast precursors (Fig. 1a−f). In Cre-negative hyperthyroid mice, trabecular bone volume of the fourth lumbar vertebra was reduced by 43.2% with a decreased trabecular number (−26.3%) and a 1.4-fold increased trabecular separation while Cre-positive mice displayed lowered bone volume by 34.9% and decreased trabecular thickness (−11.2%) with hyperthyroidism as compared to non-treated respective littermate controls (Fig. 1a−e). In addition, all hyperthyroid male mice exhibited trabecular (BV/TV Femur: Cre-negative: −48.1%, Cre-positive: −43.8% vs. respective untreated controls) and cortical bone loss at the femur (Ct.BV/TV: Cre-negative: −1.5%, Cre-positive: −1.2% vs. respective untreated controls) as compared with respective euthyroid groups (Fig. 1f, g, Table 1). Bone strength of L5 vertebrae was impaired in all hyperthyroid males (F_max_: Cre-negative: −44.8%, Cre-positive: -50.9% vs. respective untreated controls), while cortical bone strength tested at the femur was only significantly reduced in Cre-positive male mice (F_max_: Cre-negative: −13.4%, n.s., Cre-positive: −24.1% vs. respective untreated controls) (Fig. 1h, i).

**Table 1:** Body weight, femur length as well as selected bone and bone turnover parameters of male *Bmpr1a*^fl/fl^;LysM-Cre mice

Parameter	Cre-/CO *N* = 9	Cre-/T4 *N* = 8	p-value Cre-/CO vs. Cre-/T4	Cre + /CO *N* = 9	Cre + /T4 *N* = 9	*p*-value Cre + /CO vs. Cre + /T4	*p*-value Cre-/CO vs. Cre + /CO
Body weight [g]	28.4 ± 0.9	29.9 ± 1.9	0.604	30.0 ± 2.3	29.8 ± 1.9	>0.999	>0.999
Femur length [mm]	15.5 ± 0.4	15.9 ± 0.4	0.118	15.6 ± 0.5	15.9 ± 0.2	0.219	>0.999
BMD L4 [mg/cm³]	210.5 ± 22.4	131.5 ± 37.6	<0.0001	210.3 ± 20.5	163.6 ± 36.9	0.018	>0.999
BMD Femur [mg/cm³]	133.3 ± 15.9	80.9 ± 24.7	0.0003	131.7 ± 18.7	85.9 ± 30.0	0.001	>0.999
Tb.N. Femur [1/mm]	3.0 ± 0.3	2.4 ± 0.3	0.006	3.1 ± 0.2	2.6 ± 0.6	0.076	>0.999
Tb.Th Femur [mm]	0.05 ± 0.003	0.05 ± 0.003	0.103	0.05 ± 0.004	0.05 ± 0.004	>0.999	>0.999
Tb.Sp. Femur [mm]	0.34 ± 0.03	0.44 ± 0.06	0.015	0.33 ± 0.03	0.40 ± 0.09	0.098	>0.999
Ct.BMD [mg/cm³]	972.4 ± 16.9	945.4 ± 26.4	0.164	975.4 ± 18.2	949.7 ± 31.8	0.184	>0.999
Ct.Th Femur [mm]	0.18 ± 0.01	0.17 ± 0.01	0.120	0.19 ± 0.01	0.17 ± 0.02	0.013	>0.999
Ob.S/BS L4 [%]	25.0 ± 3.7	28.8 ± 5.2	0.688	27.3 ± 3.6	26.1 ± 5.3	>0.999	>0.999
Ob.N./B.Pm L4 [1/mm]	24.3 ± 4.5	29.0 ± 4.4	0.257	27.3 ± 4.2	27.1 ± 4.3	>0.999	0.978
Oc.N./B.Pm L4 [1/mm]	13.8 ± 3.1	18.3 ± 2.7	0.266	13.6 ± 5.3	19.6 ± 5.1	0.043	>0.999
MS/BS L3 [%]	14.8 ± 3.3	19.9 ± 3.9	0.079	18.8 ± 1.9	20.1 ± 5.4	>0.999	0.268
MAR L3 [µm/d]	1.7 ± 0.3	1.7 ± 0.2	>0.999	1.5 ± 0.2	1.6 ± 0.4	0.541	>0.999

Twelve-week-old male Cre-negative (Cre-) and Cre-positive (Cre +) *Bmpr1a*^fl/fl^;LysM-Cre mice were treated with L-thyroxine (T4) or received normal drinking water (CO) over 4 weeks. Micro-CT analysis of the fourth lumbar vertebra (L4), distal femur and femoral midshaft was used to determine bone mineral density (BMD), trabecular number (Tb.N), trabecular separation (Tb.Sp), trabecular thickness (Tb.Th) as well as cortical BMD (Ct.BMD) and cortical thickness (Ct.Th). Using static and dynamic histomorphometry analyses of the L4/L3 vertebra, osteoblast surface per bone surface (Ob.S/BS), osteoblast number per bone perimeter (Ob.N./B.Pm), osteoclast number per bone perimeter (Oc.N./B.Pm), mineralized bone surface per bone surface (MS/BS) and mineral apposition rate (MAR) were quantified. Mean +/- SD are indicated for each group. Statistical analysis was performed by Two-Way ANOVA.

In female cohorts, L-thyroxine treatment led to a significant increase of serum total T_4_ in both Cre-negative (6-fold) and Cre-positive (3.8-fold) mice, while total T_3_ concentrations were significantly elevated in Cre-negative (4.1-fold) but not Cre-positive mice (2.9-fold, *p* = 0.14) as compared with respective untreated controls (Fig. S2c, d). Further, female Cre-positive mice as well as hyperthyroid Cre-negative mice presented higher body weight as compared with euthyroid Cre-negative mice, however, femur length was not different between the groups (Fig. S1b, c). In line with findings in male cohorts, both Cre-negative and Cre-positive female mice showed trabecular and cortical bone loss with hyperthyroidism (Fig. S1d−k).

Bone turnover parameters P1NP and TRAP increased with hyperthyroidism in both Cre-negative and Cre-positive male and female mice, although changes in TRAP levels of euthyroid versus hyperthyroid Cre-positive mice did not reach statistical significance in the male (+31.7%, *p* = 0.056), but only female cohort (+82.3%) (Fig. 1j, k; Fig. S1l, m). Histomorphometric and histological osteoblast parameters (MS/BS, MAR, BFR/BS, Ob.S/BS, Ob.N/B.Pm) did not show significant changes in hyperthyroid males despite elevated P1NP serum levels (Fig. 1l, Table 1, Figs. S3, S4). Nevertheless, osteoclast surface and number increased by 1.6-fold and 1.4-fold, respectively, in Cre-positive hyperthyroid versus euthyroid males (Fig. 1m, Table 1, Fig. S3). Together with the raised circulating TRAP levels, these findings demonstrate a high bone resorption phenotype with hyperthyroidism for both sexes irrespective of the genotype. In contrast to the elevated TRAP serum levels with hyperthyroidism, in vitro treatment of premature osteoclasts with T_3_ until their maturation did not affect the expression of acid phosphatase 5, tartrate resistant (*Acp5*) (Fig. 1n), the gene encoding for TRAP, indicating that actions of thyroid hormones on osteoclasts might be indirectly mediated via other bone cells.

### Osteoprogenitor-specific *Bmpr1a* knockout protects hyperthyroid mice from bone loss

**Fig. 2:**
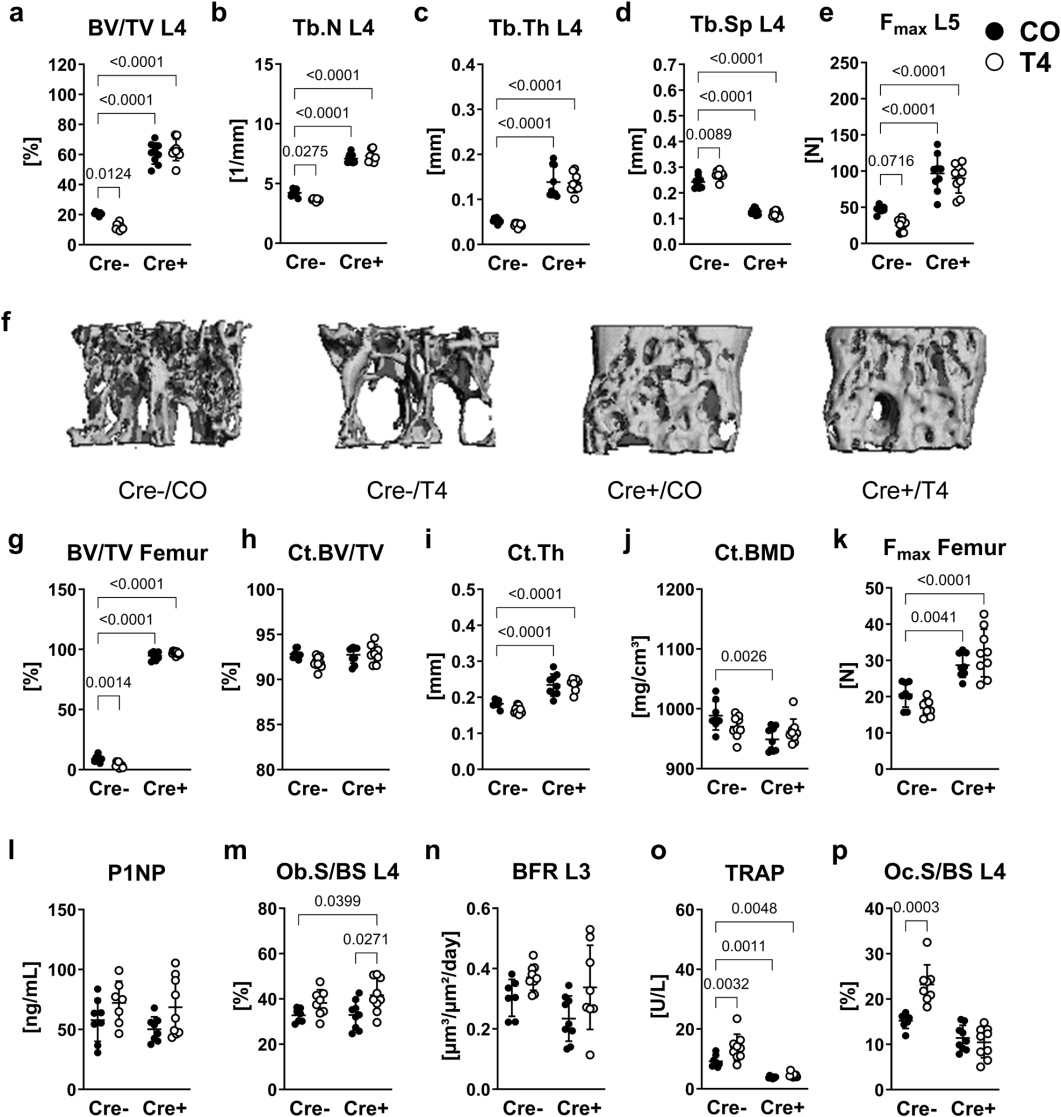
Loss of *Bmpr1a* in osteoblasts protects hyperthyroid mice from osteoporosis. Twelve-week old male Cre-negative (Cre-) and Cre-positive (Cre +) *Bmpr1a*^fl/fl^;Osx-Cre mice remained euthyroid (CO) or were rendered hyperthyroid (T4) by adding 1.2 µg/mL L-thyroxine into their drinking water over 4 weeks. Based on microCT analysis, (**a**) bone volume over total volume (BV/TV), (**b**) trabecular number (Tb.N), (**c**) trabecular thickness (Tb.Th), and (**d**) trabecular separation (Tb.Sp) were measured at the fourth lumbar vertebra (L4). **e** The maximal force (F_max_) was assessed by compression test at the L5 vertebra. **f** Representative 3D reconstructions of the trabecular compartment of L4. In addition, (**g**) trabecular BV/TV and (**h**) cortical bone volume over total volume (Ct.BV/TV), (**i**) Ct. thickness (Ct.Th) and (**j**) cortical bone mineral density (Ct.BMD) were determined at the femur. **k** The maximal force (F_max_) at the femur was tested by 3-point bending test. **l** Serum concentrations of bone formation marker P1NP was quantified using ELISA. Further, (**m**) osteoblast surface per bone surface (Ob.S/BS) and (**n**) bone formation rate per bone surface (BFR) were assessed at the spine. With regards to bone resorption, serum concentrations of (**o**) bone resorption marker TRAP and (**p**) osteoclast surface per bone surface (Oc.S/BS) were determined. Each dot indicates an individual mouse. MicroCT/Histology: Cre-/CO: *N* = 9; Cre-/T4: *N* = 9; Cre +/CO: *N* = 8; Cre +/T4: *N* = 9; ELISAs: Cre-/CO: *N* = 7; Cre-/T4: *N* = 8; Cre +/CO: *N* = 7; Cre +/T4: *N* = 8. The horizontal lines represent the mean +/- SD. Statistical analysis was performed by Two-way ANOVA and selected *p* values are shown within the graph.

Osteoblasts have been described as the primary target cells of thyroid hormones in bone^2^ and multiple osteoblast-derived cytokines and endocrine factors are known to control osteoclast development and activity, especially in the context of BMP signaling^29,30,38,39^. Therefore, we analyzed whether hyperthyroid mice with an osteoprogenitor-specific *Bmpr1a* deletion might be protected against bone loss. As described before^32^, conditional *Bmpr1a* knockout in osteoprogenitors per se led to an increase of trabecular bone volume (L4: males: 2.9-fold, females: 6.5-fold), trabecular number (L4: males: 1.7-fold, females: 2.2-fold), trabecular thickness (L4: males: 2.6-fold, females: 3.4-fold), a reduction of trabecular separation (L4: males: −47.2%, females: −58.7%) and at the spine (Fig. 2a−d, f) and femur in male (Fig. 2g, Table 2) and female mice (Fig. S5) as compared to respective Cre-negative littermates. Furthermore, Cre-positive *Bmpr1a*^fl/fl^;Osx-Cre males presented improved bone strength (L5: 2.1-fold; femur: 1.4-fold versus untreated respective controls, Fig. 2e, k). Femur length, body weight and cortical bone volume were not affected by conditional *Bmpr1a* knockout, however, we observed thickened cortices (males: +28.6%, females: +29.7% vs. untreated Cre-negative mice) and decreased cortical bone mineral density (males: −2.9%, females: −4.0% vs. untreated Cre-negative mice), as previously described^40^, in male and female mice lacking *Bmpr1a* in osteoprogenitors (Table 2, Fig. 2h−j, Fig. S3n−p).

**Table 2:** Body weight, femur length as well as selected bone and bone turnover parameters of male *Bmpr1a*^fl/fl^;Osx-Cre mice

Parameter	Cre-/CO *N* = 8	Cre-/T4 *N* = 9	*p* value Cre-/CO vs. Cre-/T4	Cre + /CO *N* = 9	Cre + /T4 *N* = 9	*p* value Cre + /CO vs. Cre + /T4	*p* value Cre-/CO vs. Cre + /CO
Body weight [g]	28.8 ± 0.7	29.7 ± 0.3	>0.999	26.5 ± 0.7	29.2 ± 0.7	**0.039**	0.164
Femur length [mm]	15.0 ± 0.4	15.5 ± 0.4	>0.999	15.1 ± 0.2	15.4 ± 0.5	>0.999	>0.999
BMD L4 [mg/cm³]	219.0 ± 31.3	143.6 ± 17.1	0.006	568.9 ± 52.6	598.1 ± 56.1	0.940	<0.0001
BMD Femur [mg/cm³]	121.4 ± 34.7	69.1 ± 24.5	0.008	914.2 ± 41.3	966.1 ± 18.6	0.009	<0.0001
Tb.N. Femur [1/mm]	3.4 ± 0.7	2.6 ± 0.5	0.060	5.9 ± 0.6	5.6 ± 0.8	>0.999	<0.0001
Tb.Th Femur [mm]	0.05 ± 0.001	0.04 ± 0.009	>0.999	0.32 ± 0.05	0.35 ± 0.05	0.440	<0.0001
Tb.Sp. Femur [mm]	0.31 ± 0.06	0.42 ± 0.10	0.011	0.07 ± 0.02	0.06 ± 0.02	>0.999	<0.0001
Ob.N./B.Pm L4[1/mm]	29.3 ± 3.3	35.7 ± 5.5	0.164	30.5 ± 6.5	36.9 ± 5.3	0.104	>0.999
Oc.N./B.Pm L4 [1/mm]	9.3 ± 1.5	13.1. ± 2.2	0.0013	7.9 ± 1.7	6.8 ± 1.8	>0.999	0.813
MS/BS L3 [%]	20.8 ± 2.6	20.8 ± 1.7	>0.999	14.7 ± 3.1	20.1 ± 5.8	0.143	0.007
MAR L3 [µm/d]	1.4 ± 0.2	1.7 ± 0.2	>0.999	1.6 ± 0.5	1.9 ± 0.6	0.80	>0.999

Twelve-week-old male Cre-negative (Cre-) and Cre-positive (Cre +) *Bmpr1a*^fl/fl^;Osx-Cre mice were treated with L-thyroxine (T4) or received normal drinking water (CO) over 4 weeks. Micro-CT analysis of the fourth lumbar vertebra (L4), distal femur and femoral midshaft was used to determine bone mineral density (BMD), trabecular number (Tb.N), trabecular separation (Tb.Sp), and trabecular thickness (Tb.Th). Based on static and dynamic histomorphometry analyses of the L4/L3 vertebra, osteoblast surface per bone surface (Ob.S/BS), osteoblast number per bone perimeter (Ob.N./B.Pm), osteoclast number per bone perimeter (Oc.N./B.Pm), mineralized bone surface per bone surface (MS/BS) and mineral apposition rate (MAR) were quantified. Mean +/- SD are indicated for each group. Statistical analysis was performed by Two-Way ANOVA.

Serum concentrations of total T_4_ (Male Cre-negative: 4.8-fold; male Cre-positive: 4.7-fold; female Cre-negative: 5.1-fold; female Cre-positive: 3.1-fold vs respective untreated controls) and total T_3_ (Male Cre-negative: 2.4-fold; male Cre-positive: 2.1-fold; female Cre-negative: 3.9-fold; female Cre-positive: 1.9-fold vs respective untreated controls) increased with L-thyroxine treatment in all groups (Fig. S2e−h). With hyperthyroidism, Cre-negative but not Cre-positive male mice developed an osteoporotic phenotype as shown by lowered trabecular bone volume (−56.8%), abated trabecular number (−13.5%) and increased trabecular separation (+11.8%) at the L4 as compared with untreated respective controls (Fig. 2a−e). Bone strength showed a tendency to decrease by −47.3% (*P* = 0.072) only in spines of Cre-negative hyperthyroid males as compared to untreated controls (Fig. 2e). In femurs, we found decreased trabecular bone volume (−59.4% vs untreated, Cre-negative mice), low bone mineral density and enhanced trabecular separation with hyperthyroidism in Cre-negative mice (Fig. 2g, Table 2) and an even increased bone mineral density in hyperthyroid conditional knockout mice as compared with respective, untreated controls (Table 2). Cortical bone volume, thickness, bone mineral density and bone strength were not altered by hyperthyroidism in either wildtype mice or mice lacking *Bmpr1a* in osteoprogenitors (Fig. 2h−k).

With regards to bone formation, circulating levels of P1NP, osteoblast surface, and osteoblast numbers as well as MAR and BFR showed a tendency towards increase in Cre-negative male mice with L-thyroxine treatment as compared with respective untreated mice (Fig. 2l−n, Table 2, Fig. S6). In Cre-positive males osteoblast surface increased with hyperthyroidism by 1.3-fold (Fig. 2m; Fig. S7), while again other osteoblast parameters demonstrated a tendency towards increase (Fig. 2l, n, Table 2). Of note, calcein labels in bones from Cre-positive *Bmpr1a*^fl/fl^;Osx-Cre males showed an overall low fluorescence signal, while T_4_ treatment enhanced the fluorescence intensity resulting in wide, but often not clearly separable fronts of bone formation as shown by the representative pictures in the Supplementary Figs. (Fig. S6).

Nevertheless, only hyperthyroid Cre-negative mice presented increased serum levels of bone resorption marker TRAP (+1.5-fold vs. untreated Cre-negative mice), enlarged osteoclast surfaces (1.5-fold vs. untreated Cre-negative mice) and an elevated osteoblast number, while conditional knockout mice presented low TRAP serum concentrations in general (Fig. 2o, p, Table 2, Fig. S7).

In female mice, we observed weight gain with hyperthyroidism in Cre-negative (+13.9% vs. untreated, Cre-negative mice) and Cre-positive cohorts (+10.3% vs. untreated, Cre-positive mice), while femur length was not different between the four groups (Fig. S5a, b). At the spine, no alterations of bone parameters due to thyroid hormone excess were detected in both wildtypes and conditional knockouts (Fig. S5c−h). Nevertheless, decreased trabecular number (−36.8% vs. untreated, Cre-negative mice, Fig S5k) and augmented trabecular separation (+44.3% vs. untreated, Cre-negative mice, Fig. S5m) at th femur and upregulated serum levels of both P1NP (+60.5% vs. untreated, Cre-negative mice, Fig. S5q) and TRAP (+65.9% vs. untreated, Cre-negative mice, Fig. S5r) indicate an early stage of hyperthyroidism-driven bone loss in female hyperthyroid Cre-negative mice, but not mice lacking *Bmpr1a* in osteoprogenitors.

Thus, we identified the osteoblastic BMP receptor *Bmpr1a* as a critical contributor to hyperthyroidism-driven high bone resorption in male and female mice.

### BMP receptor BMPR1A mediates thyroid hormone actions in osteoblasts and regulates osteoblast-osteoclast interactions

**Fig. 3:**
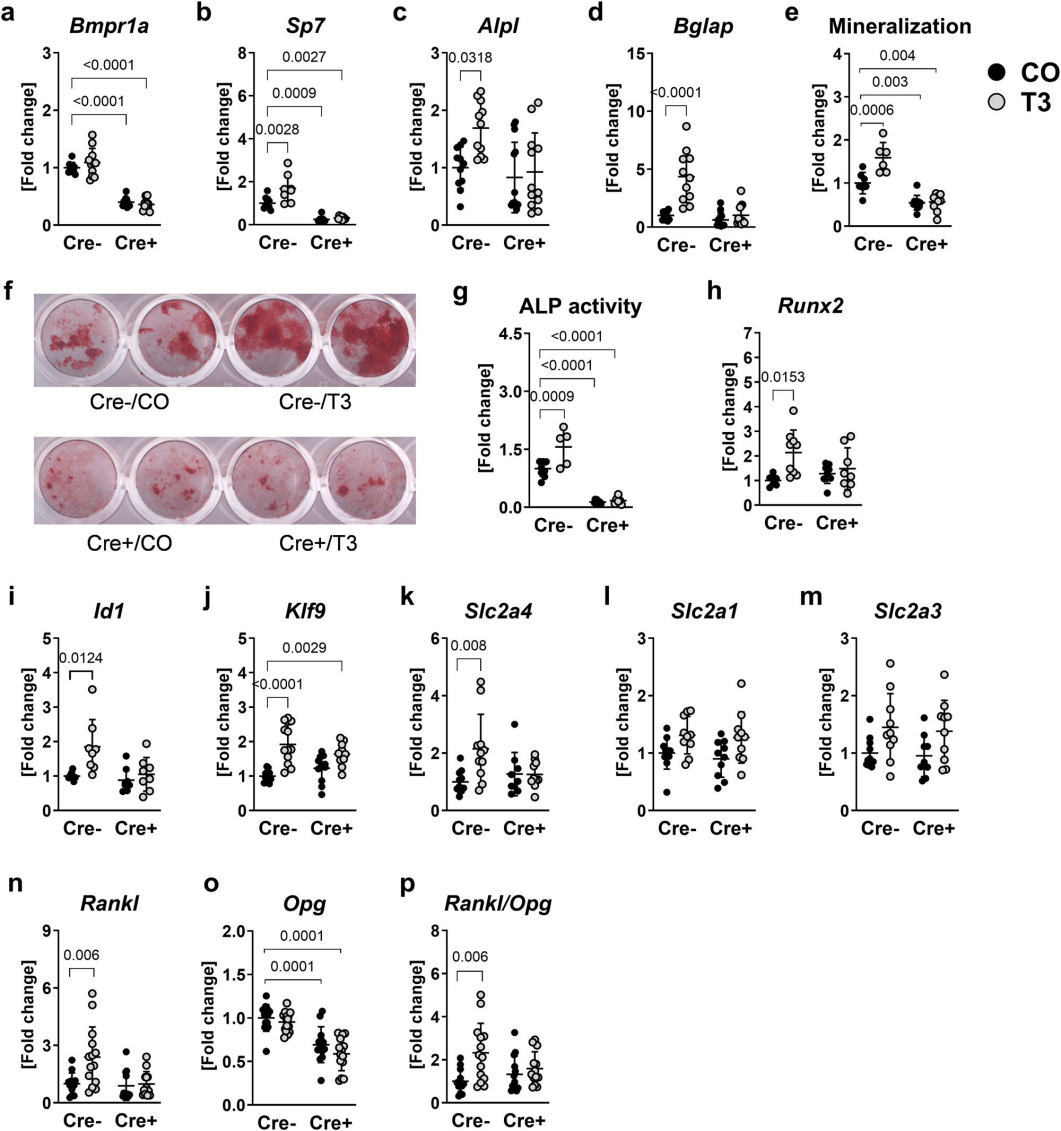
BMPR1A mediates the response to T_3_ in osteoblasts in vitro. Using quantitative real-time PCR analysis, mRNA expression of (**a**) *Bmpr1a*, (**b**) osterix (*Sp7*), (**c**) alkaline phosphatase (*Alpl*) and (**d**) osteocalcin (*Bglap*) was measured in primary murine osteoblasts derived from *Bmpr1a*^fl/fl^;Osx-Cre mice after 48 h treatment with 100 nM T_3_ (T3). Further, (**e**) mineralization capacity was evaluated after differentiation and T_3_-treatment over 10 days. **f** Representative image of Alizarin Red stained bone matrix. **g** ALP activity was assessed after 72 h treatment with T_3_. In addition, transcript levels of BMP target genes (**h**) runt related transcription factor 2 (*Runx2*) and (**i**) inhibitor of DNA binding 1 (*Id1*), thyroid hormone target gene (**j**) Krueppel-like factor 9 (*Klf9*), glucose transporters (**k**) *Slc2a4* (**l**) *Slc2a1* and (**m**) *Slc2a3*, (**n**) receptor activator of NF-κB ligand (*Rankl*), and (**o**) osteoprotegerin (*Opg*) were quantified and the (**p**) *Rankl/Opg* ratio was calculated. Each dot indicates an individual mouse. Real-time PCR: Cre-/CO: *N* = 8-11; Cre-/T3: *N* = 8-14; Cre +/CO: *N* = 11-13; Cre +/T3: *N* = 8-13; Mineralization: Cre-/CO: *N* = 7; Cre-/T3: *N* = 6; Cre +/CO: *N* = 8;Cre +/T3: *N* = 8; ALP assay: Cre-/CO: *N* = 8; Cre-/T3: *N* = 5; Cre +/CO: *N* = 9;Cre +/T3: *N* = 5. The horizontal lines represent the mean +/- SD. Statistical analysis was performed by Two-way ANOVA and selected *p* values are shown within the graph.

To investigate how the thyroid hormone response in osteoblasts is altered by *Bmpr1a* knockout, we cultured osteoblasts derived from Cre-negative and Cre-positive *Bmpr1a*^fl/fl^;Osx-Cre mice and treated them with pharmacological doses of T_3_ that is known to have a 10-fold higher binding affinity to thyroid hormone receptors than T_4_^2^. *Osterix*-promotor driven *Bmpr1a* deletion led to a 59.9% decrease of *Bmpr1a* expression in mature osteoblasts as compared to wildtype osteoblasts (Fig. 3a). T_3_ treatment increased the expression of prominent osteoblast marker genes *Osx*, alkaline phosphatase (*Alpl*) and osteocalcin (*Bglap*) (1.7-fold, 1.7-fold, 4.3-fold vs. untreated, respective control) as well as improved mineralization capacity and ALP activity (both 1.6-fold vs. untreated, respective control) in wildtype osteoblasts (Fig. 3b−g). *Bmpr1a*-lacking osteoblasts presented downregulated *Osx* expression (−75.2% vs. untreated, respective control), lowered mineralization capacity (−45.6% vs. untreated, respective control) and decreased ALP activity (−86.3% vs. untreated, respective control) and were not affected by T_3_ treatment (Fig. 3b−g).

While expression of BMP target genes *Runx2* and *Id1* and thyroid hormone target gene *Klf9* were not altered with T_3_ in *Bmpr1a* knockout osteoblasts, their T_3_-dependent regulation in wildtype osteoblasts indicates that the transcriptional activation of both pathways relies on *Bmpr1a* expression (Fig. 3h−j). As a link to increased energy demands with hyperthyroidism, we checked the expression of *Slc2a1*, *Slc2a3* and *Slc2a4* encoding for class I glucose transporters GLUT1, GLUT3 and GLUT4, respectively, that facilitate glucose transport across the cell membrane. Interestingly, we found 2.2-fold elevated levels of *Slc2a4*, the gene coding for the primary glucose transporter GLUT4 in osteoblasts^41^, in T_3_-treated wildtype but not *Bmpr1a* knockout osteoblasts as compared to respective untreated controls (Fig. 3k). Expression of *Slc2a1* and *Slc2a3* tended to increase in both T_3_-treated groups, but did not reach statistical significance (*Slc2a1:* Cre-/T_3_: 1.3-fold, *P* = 0.27; Cre +/T_3_: 1.4-fold, *P* = 0.14; *Slc2a3:* Cre-/T_3_: 1.5-fold, *P* = 0.18; Cre +/T_3_: 1.5-fold, *P* = 0.29 vs. respective untreated control) (Fig. 3l, m).

**Fig. 4:**
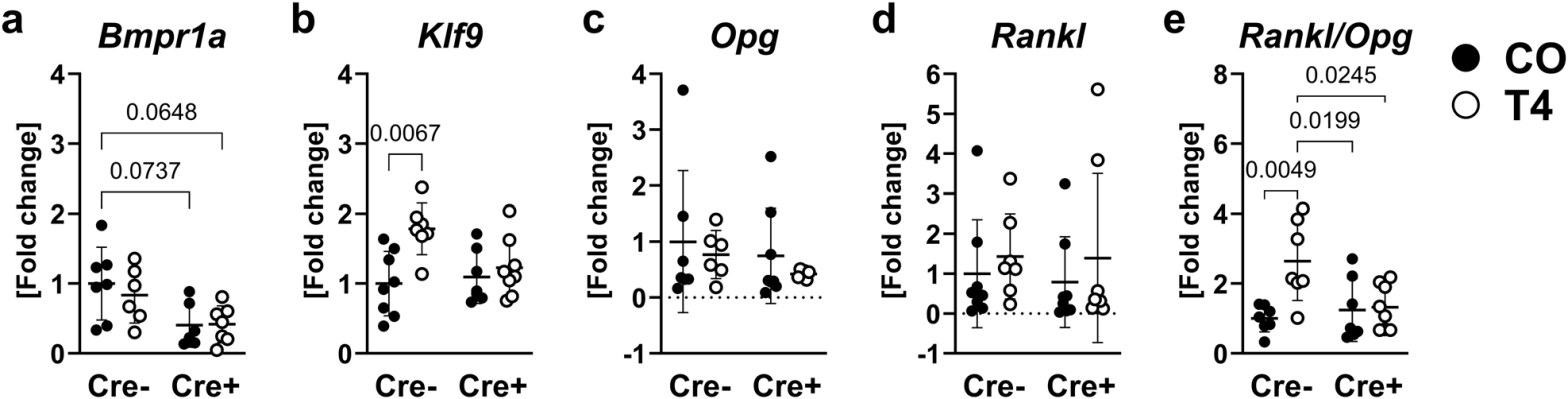
BMPR1A controls the *Rankl/Opg* ratio in response to T_4_ in vivo. Twelve-week old male Cre-negative (Cre-) and Cre-positive (Cre +) *Bmpr1a*^fl/fl^;Osx-Cre mice remained euthyroid (CO) or were rendered hyperthyroid (T4) by adding 1.2 µg/mL L-thyroxine into their drinking water over 4 weeks. Total RNA was isolated from bone tissue and the expression of (**a**) *Bmpr1a*, (**b**) *Klf9*, (**c**) *Opg* and (**d**) *Rankl* was evaluated using quantitative real-time PCR. Further, (**e**) the ratio of *Rankl* and *Opg* was calculated. Each dot indicates an individual mouse. Cre-/CO: *N* = 8; Cre-/T4: *N* = 7; Cre +/CO: *N* = 8; Cre +/T4: *N* = 8. The horizontal lines represent the mean +/- SD. Statistical analysis was performed by Two-way ANOVA and selected *p* values are shown within the graph.

Further, we showed that *Rankl* was upregulated in T_3_-treated wildtype osteoblasts (2.4-fold vs. untreated respective control) and expression of *Opg* was downregulated in both untreated and T_3_-treated knockout osteoblasts (CO: −30.7%, T_3_: −41.3%) as compared with non-treated wildtype cells, resulting in 2.3-fold increased *Rankl/Opg* ratio only in thyrotoxic wildtype but not *Bmpr1a*-deficient osteoblasts (Fig. 3n−p). In bone tissue of *Bmpr1a*^fl/fl^;Osx-Cre mice, *Bmpr1a* expression tended to decrease in mice with active Cre-recombinase (−59.4%, *P* = 0.07) (Fig. 4a) and *Klf9* expression was upregulated by 78.4% with hyperthyroidism (Fig. 4b) as compared with Cre-negative, euthyroid mice. While transcript levels of *Rankl* and *Opg* were not different between the four groups, *Rankl/Opg* ratio again was 2.6-fold increased with hyperthyroidism in bone tissue obtained from wildtype mice only, supporting our in vitro findings (Fig. 4c−e).

**Fig. 5:**
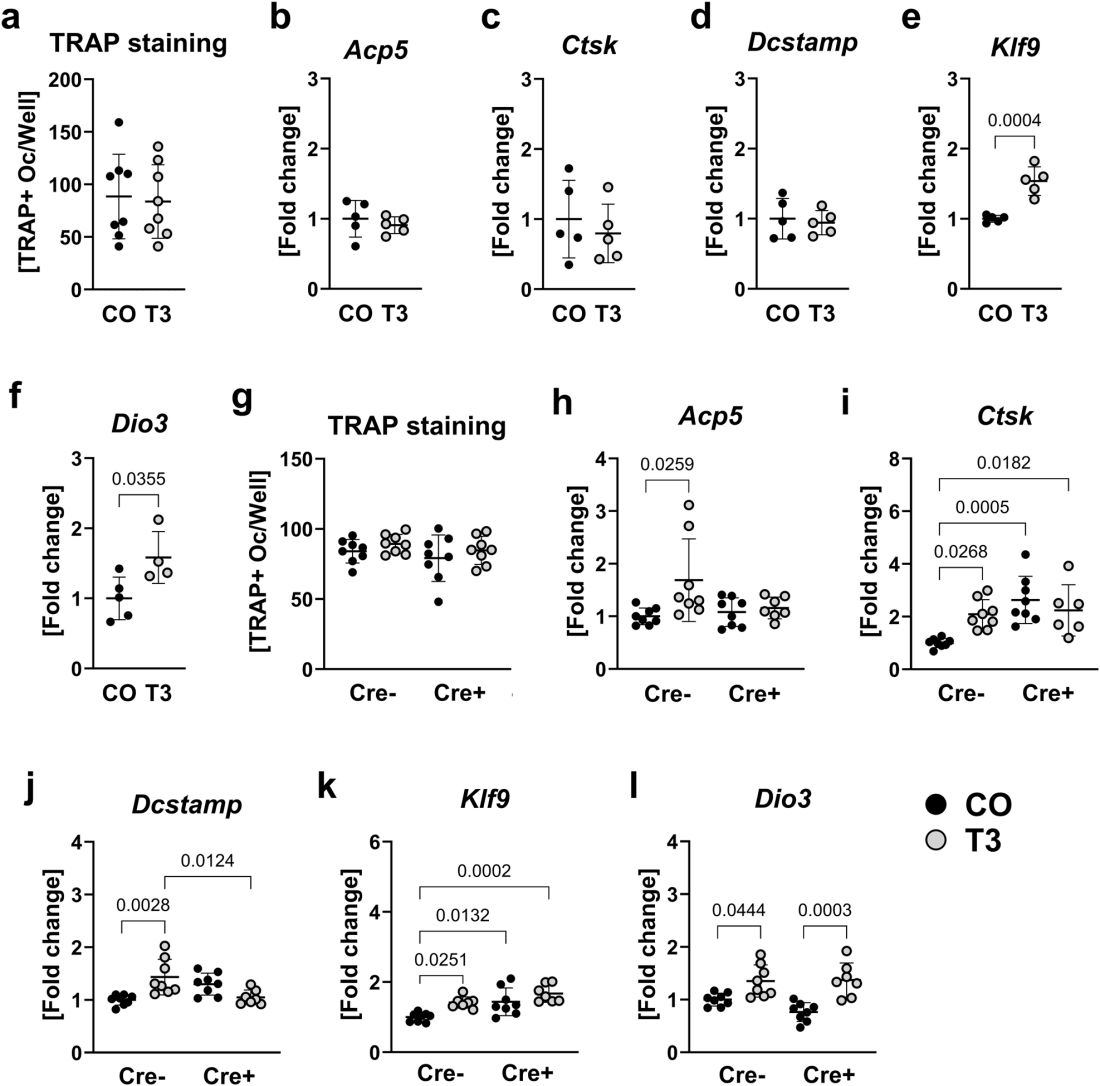
Direct versus indirect actions of T_3_ on primary wildtype osteoclasts. Early wildtype osteoclasts (d4) were treated directly with 100 nM T_3_ over 48 h (**a**−**f**) and (**a**) number of TRAP-positive multinucleated osteoclasts (≥3 nuclei) was counted after TRAP staining. In addition, expression of osteoclast marker genes (**b**) acid phosphatase 5, tartrate resistant (*Acp5*), (**c**) cathepsin K (*Ctsk*), and (**d**) dendrocyte expressed seven transmembrane protein (*Dcstamp*) as well as thyroid hormone target genes (**e**) krueppel-like factor 9 (*Klf9*) and (**f**) deiodinase 3 (*Dio3*) was quantified. Furthermore, early wildtype osteoclasts (d4) were treated over 48 h with supernatants from untreated (CO) or T_3_-treated (T3) Cre-negative (Cre-) and Cre-positive (Cre +) *Bmpr1a*^fl/fl^;Osx-Cre osteoblasts (**g**−**l**). Subsequently, (**g**) number of TRAP-positive multinucleated osteoclasts was evaluated as well as the expression of (**h**) *Acp5*, (**i**) *Ctsk*, (**j**) *Dcstamp*, (**k**) *Klf9*, and (**l**) *Dio3*. Each dot indicates an individual mouse. TRAP staining (**a**, **g**): *n* = 8 per group. RNA expression analysis: direct treatment (**b**−**f**): *n* = 5 per group. Indirect coculture (**h**−**l**): Cre-/CO: *N* = 8; Cre-/T3: *N* = 8; Cre +/CO: *N* = 8; Cre +/T3: *N* = 7. The horizontal lines represent the mean +/- SD. Statistical analysis was performed by a two-sided student´s *t* test comparing two groups or a two-way ANOVA for the comparison of four groups. Significant *p* values are shown within the graph.

Given that RANKL and OPG are soluble factors secreted by osteoblasts, we used osteoblast supernatants for the treatment of wildtype osteoclasts to investigate whether thyroid hormones can indirectly affect osteoclastogenesis and whether this effect might be BMPR1A-dependent. A direct treatment of early osteoclasts with T_3_ did not alter the number of TRAP-positive osteoclasts or the expression of common osteoclast marker genes (Fig. 5a−d). Interestingly, thyroid hormone target genes *Klf9* and deiodinase 3 (*Dio3*) were upregulated 1.5-fold and 1.6-fold, respectively, suggesting that osteoclasts do respond to thyroid hormone treatment, however, this did not translate into phenotypic changes (Fig. 5e, f). Despite osteoclast numbers not being affected by supernatant treatment, expression of osteoclast marker genes *Acp5* (1.7-fold), cathepsin K (*Ctsk*, 2.1-fold), and dendrocyte expressed seven transmembrane protein (*Dcstamp*, 1.4-fold) as well as *Klf9* (1.4-fold) and *Dio3* (1.4-fold) increased when osteoclasts were challenged with supernatants derived from T_3_-treated Cre-negative osteoblasts as compared to untreated Cre-negative cells (Fig. 5g−l). In contrast comparing the treatment with supernatants derived from untreated versus T_3_-treated Cre-positive osteoblasts, only the expression of *Dio3* was significantly upregulated by 1.8-fold indicating that BMPR1A is involved in osteoblast-osteoclast interactions under thyrotoxic conditions (Fig. 5l).

In conclusion, we show that the BMP receptor BMPR1A is an important mediator of thyroid hormone actions in osteoblasts and regulates the *Rankl/Opg* ratio in osteoblasts and bone tissue in response to thyroid hormones, thus potentially promoting osteoclastogenesis and bone resorption.

## Discussion

Hyperthyroidism is an established cause of osteoporosis, still, the underlying molecular mechanisms are not fully understood yet. In our previous study, we showed that thyroid hormones activate BMP signaling in osteoblasts and that, in vivo, blocking BMP signaling using a BMPR1A-specific ligand scavenger prevented bone loss in hyperthyroid mice^26^. Nevertheless, this systemic approach did not reveal which bone cell type might be the main driver of hyperthyroidism-induced osteoporosis.

Given the high bone resorption observed with thyroid hormone excess, we first analyzed the bone phenotype of mice lacking *Bmpr1a* in osteoclast progenitors. In contrast to a histomorphometry-based study showing mild trabecular bone gain in femurs of 8 to 10-week-old *Bmpr1a*^fl/fl^;LysM-Cre-positive versus control mice^42^, we did not observe any significant skeletal alterations using microCT analysis or changes in serum bone turnover markers at an age of 16 weeks in male and female conditional knockout mice. Cathepsin K (Ctsk)-promotor-driven *Bmpr1a* knockout targeting mature osteoclasts, however, leads to marked trabecular bone gain due to enhanced bone formation at a young age implying that BMPR1A mediates osteoclast-to-osteoblast coupling through mature osteoclasts mainly^36^. In vitro analysis of osteoclasts lacking *Bmpr1a* based on LysM-Cre versus Ctsk-Cre driven deletion showed again contradictory results with reduced number of TRAP-positive osteoclasts and low *Acp5* expression^42^ or no effect on *Acp5* expression in *Bmpr1a*^fl/fl^LysM-Cre-positive cells, while osteoclastogenesis increased with Ctsk-Cre-based *Bmpr1a* knockout. Thus, the timing of *Bmpr1a* deletion during osteoclast development seems to be critical and primarily differentiated osteoclasts are affected by disrupted BMP signaling.

With hyperthyroidism, *Bmpr1a*^fl/fl^;LysM-Cre mice presented osteoporotic bones with impaired bone strength independent of osteoclast progenitor-specific *Bmpr1a* deletion, indicating that either BMPR1A plays only a minor role mediating effects of thyroid hormones on osteoclastogenesis and osteoclast-osteoblast coupling or that osteoclasts per se are not directly responsive to thyroid hormone treatment. In line, we could not observe any changes in *Acp5* expression in either knockout or wildtype osteoclasts when treated with T_3_. Several studies show that thyroid hormone actions on osteoclasts and bone resorption are indirectly mediated via cells of the osteoblast lineage^2,21–26^ corroborating our findings of direct versus indirect thyroid hormone treatment of wildtype osteoclasts. In contrast to our previous studies^26,43,44^ and the results from the *Bmpr1a*^fl/fl^;Osx-Cre cohorts, only circulating P1NP levels but not histomorphometric osteoblast parameters increased with L-thyroxine treatment in *Bmpr1a*^fl/fl^;LysM-Cre mice. Still, osteoclast numbers showed a tendency to increase or were significantly enhanced in hyperthyroid Cre-negative and Cre-positive *Bmpr1a*^fl/fl^;LysM-Cre mice, respectively, suggesting together with elevated TRAP serum levels high bone resorptive activities under thyroid hormone excess regardless of *Bmpr1a* expression in osteoclast progenitors.

Given that loss of *Bmpr1a* expression in osteoclasts did not prevent hyperthyroidism-driven bone loss, we next focused on osteoblasts. While osteoblast-specific *Bmpr1a* deletion led to a osteosclerotic bone phenotype as described before^32^, thyroid hormone treatment did not promote bone resorption or bone loss in male and female *Bmpr1a*^fl/fl^;Osx-Cre-positive mice revealing an important role of osteoblastic BMPR1A in the pathogenesis of hyperthyroidism-induced bone loss and fragility.

Consistently, besides more general approaches such as blocking BMP type I receptors or scavenging BMP ligands using the inhibitor LDN193189 and anti-BMP ligand antibodies, respectively^26^, also targeted genetic *Bmpr1a* knockout hampered the response of osteoblasts to T_3_ treatment in vitro. In particular, mineralization capacity, ALP activity and expression of the early osteoblast marker osterix were impaired with *Bmpr1a* loss, in line with findings in osteoblasts derived from *Bmpr1a*^fl/fl^;Og2-Cre^45^, *Bmpr1a*^fl/fl^;Prx1-CreERT^46^ and *Bmpr1a*^fl/fl^;Col1-ERT^TM^-Cre^47^ mice, and were not enhanced with T_3_ in contrast to osteoblasts expressing *Bmpr1a*. Further, we show that not only the expression of BMP target genes (*Runx2*, *Id1*), but also the thyroid hormone target gene *Klf9* was not induced in T_3_-treated *Bmpr1a*-deficient osteoblasts and bone tissue from hyperthyroid *Bmpr1a*^fl/fl^;Osx-Cre-positive mice, respectively, allowing for speculations on close mutual interactions between components of thyroid hormone signaling and the BMP signaling pathway.

Dynamic histomorphometric analysis revealed overall low fluorescence signals from calcein labels in untreated Cre-positive *Bmpr1a*^fl/fl^;Osx-Cre animals suggesting a slow bone formation in these mice (Fig. S6) as reported before in mouse models with Col1- or Og2-driven *Bmpr1a* deletion^29,45^. T_4_ treatment increased the overall fluorescence intensity of the labels, however, a detection of clearly separated double labels was difficult (Fig. S6). In addition, enlarged osteoblast surface and a tendency to increase of other osteoblast parameters (Ob.N, P1NP) indicate that, in contrast to our in vitro findings, *Bmpr1a*-lacking osteoblasts are still stimulated by thyroid hormone treatment in vivo. However, within the bone marrow and next to osteoblasts, several other cell types such as hematopoietic cells and marrow adipocytes reside and were shown to also be affected by thyroid hormones^48–52^. Thus, we cannot exclude indirect stimulation of the *Bmrp1a*-depleted osteoblasts by other surrounding cells and further investigations are needed to estimate the extent of these cell-cell interaction-based effects in our hyperthyroidism mouse model.

High osteoblast activity due to thyroid hormone excess requires high amounts of energy, likely in the form of glucose^53,54^. Osteoblasts utilize glucose primarily via aerobic glycolysis, a tightly regulated process that can generate adenosine triphosphate quickly^53,54^. Glucose uptake is mainly performed by class I glucose transporters, GLUT1, -3 and -4^41^. GLUT2 expression was not detected in osteoblasts^41^. Based on in vitro *Slc2a* knockout studies, especially *Slc2a4* expression was shown to be crucial for osteoblast proliferation and differentiation, albeit also other GLUTs might contribute to a balanced glucose uptake and thus, be necessary to cover the energy needed for new bone formation^41^. A previous study confirmed that T_3_ stimulates glucose uptake via glucose transporters GLUT1 and GLUT3 into osteoblasts in vitro, however, GLUT4 was not investigated in that study^55^. Vice versa, we have previously shown that hypothyroidism leads to reduced glucose uptake and correspondingly low *Slc2a4* expression in bone tissue in mice^56^. Linking glucose metabolism to BMP signaling, BMPR1A was reported to regulate the expression of glucose transporter *Slc2a1* in chondrocytes in vivo^57^ and its BMP ligand BMP2 was shown to upregulate *Slc2a4* expression in adipocytes^58^. In this study, we observed increased expression of *Slc2a4* in osteoblasts with hyperthyroidism in vitro indicating possibly elevated glucose uptake due to higher energy demands in thyroid hormone-stimulated osteoblasts, an effect that was blocked in *Bmpr1a*-deficient cells. Thus, *Bmpr1a* loss could hamper the glucose uptake needed to meet high energy demands and might disrupt osteogenic thyroid hormone response not only at a transcriptional but also metabolic level. Further in-depth metabolic analyses are needed to unravel the effects of thyroid hormones on metabolic processes and energy levels in osteoblasts in detail, also considering the different states of osteogenic differentiation with distinct needs for energy and the role of the BMP signaling pathway therein.

As shown before^30,39,46,59^, BMPR1A is not only critical for osteogenic differentiation, but also regulates the expression of *Rankl* and *Opg* in osteoblasts to promote osteoclastogenesis and thus, couples bone formation and bone resorption via RANKL forward signaling. In response to thyroid hormones, wildtype, but not *Bmpr1a*-deficient osteoblasts upregulated *Rankl* expression in vitro and the *Rankl/Opg* ratio increased in both Cre-negative osteoblasts and bone tissue. Loss of osteoblastic *Bmpr1a* inhibited the thyroid hormone-driven increase of the *Rankl/Opg* ratio and thus, potentially prevented high bone resorption and bone loss in hyperthyroid mice. Corroborating our hypothesis, osteoblast-mediated but not direct treatment of wildtype osteoclasts with T_3_ promoted the expression of osteoclast marker genes in a BMPR1A-dependent manner. In contrast to the in vivo setting, osteoclast numbers were not affected suggesting that additional factors such as cell-cell-interactions might play an important role. As such, RANKL reverse signaling via vesicular RANK secreted by maturing or apoptotic osteoclasts binding to membranous RANKL on osteoblast surfaces was shown to upregulate *Runx2* expression and promote osteogenic differentiation and bone formation^60,61^. Thus, BMPR1A-induced *Rankl* expression could be an important coupling factor between bone formation and bone resorption in both ways and loss of *Bmpr1a* might disrupt their fine-tuned balance in bone remodeling. Furthermore, thyroid hormone-induced *Rankl* expression in osteoblasts might start an amplification loop between osteoblasts and osteoclasts resulting in high bone turnover-induced bone loss that can be prevented by *Bmpr1a* deletion in osteoblasts.

Besides its strength, our study may have potential limitations. Aside from the great value of the Cre/LoxP system to investigate genes of interest in a cell/tissue-specific manner, possible off-targeting need to be considered. As such, LysM-Cre can target not only monocytes and macrophages, but also neutrophils^62^ and Osx-Cre can target also additional cell populations such as osteocytes, hypertrophic chondrocytes, stromal cells, adipocytes, and perivascular cells of bone marrow^63^. Beside osteoblasts, also osteocytes are crucial for orchestrating bone remodeling by secreting RANKL^64^, can import thyroid hormones^16^ and have been shown to exhibit resorptive TRAP activity themselves in response to hyperthyroidism in vivo^65^. Osteocytes are not the main target cells for the Osx-Cre-driven recombination^66^, but are the dominant cells type (90−95%) within bone tissue^67^. In line, we observed only a tendency to decreased *Bmpr1a* expression in bone tissue derived from *Bmpr1a*^fl/fl^;Osx-Cre positive mice (Fig. 4a) and thus, results based on total RNA from bone tissue should be interpreted carefully. Whether osteocytes directly contribute to thyroid hormone excess-driven osteoclastic bone resorption in a BMPR1A-dependent manner needs to be investigated in further studies using osteocyte-targeting Cre-lines. Furthermore, other signaling pathway besides BMP signaling might be activated by thyroid hormones in osteoblasts and thus, unbiased approaches such as RNA sequencing or proteomic profiling are necessary to identify further molecular changes that can drive thyroid hormone responses in bone cells.

In conclusion, this study is the first to reveal the importance of the osteoblastic BMP receptor BMPR1A in the pathogenesis of hyperthyroidism-induced osteoporosis by coupling thyroid hormone-stimulated osteoblast activity to high bone resorption potentially via the RANKL/OPG system.

## Methods

### Animal models

Floxed *Bmpr1a* mice on C57BL/6 background that carry loxP sites flanking exon 4 of the gene *Bmpr1a* (*Bmpr1a*^fl/fl^) were a kind gift from Prof. Yuji Mishina (University of Michigan, MI, USA)^68^. For generating osteoblast-specific *Bmpr1a*-deficient animals, *Bmpr1a*^fl/fl^ mice were crossed with doxycycline-repressible Osterix-Cre (Osx-Cre) mice on a C57BL/6 background^69^. Due to skeletal alterations seen in young Osx-Cre mice even without floxed alleles^70^, it is advised to repress Cre-recombinase activity prenatally up to the first 4−5 weeks of postnatal development^71,72^. Therefore, breeding pairs of the generated *Bmpr1a*^fl/fl^;Osx-Cre mice as well as their offspring received a 10 mg/mL doxycycline dissolved in 3% sucrose solution in their drinking water *ad libitum* up to 5 weeks of age. In addition, *Bmpr1a*^fl/fl^;LysM-Cre were generated to inactivate *Bmpr1a* specifically in osteoclast progenitors by crossing *Bmpr1a*^fl/fl^ mice with LysM-Cre mice on a C57BL/6 background^73^. Efficient *Bmpr1a* deletion and recombination in Cre-targeted cells and organs of Cre-positive animals was confirmed by PCR analysis^68^. Respective age-matched Cre-negative littermates were used as controls. All mice were on a C57BL/6 background. Mice were maintained in groups up to four animals in a light-dark cycle of 12/12 h at room temperature in filter-top cages with cardboard houses for enrichment purposes and had *ad libitum* access to their respective drinking water and standard chow diet.

To induce hyperthyroidism, 12-week-old Cre-negative (Cre-) and Cre-positive (Cre +) male and female mice were treated over 4 weeks with 1.2 µg/ml L-thyroxine (T4, Sigma-Aldrich, Munich, Germany) in their drinking water. Control littermates received regular tap water (CO). Group sizes were as follows: *Bmpr1a*^fl/fl^;Osx-Cre mice: Males: Cre-/CO: *N* = 8; Cre-/T4: *N* = 9; Cre +/CO: *N* = 9;Cre +/T4: *N* = 9; Females: Cre-/CO: *N* = 9; Cre-/T4: *N* = 7; Cre +/CO: *N* = 9;Cre +/T4: *N* = 8; *Bmpr1a*^fl/fl^;LysM-Cre mice: Males: Cre-/CO: *N* = 9; Cre-/T4: *N* = 9; Cre +/CO: *N* = 8;Cre +/T4: *N* = 9; Females: Cre-/CO: *N* = 7; Cre-/T4: *N* = 6; Cre +/CO: *N* = 8;Cre +/T4: *N* = 8.

At an age of 16 weeks, all mice were euthanized using CO_2_. Blood was collected via heart puncture and serum was obtained by centrifugation. The fourth lumbar vertebrae (L4) and femurs were collected postmortem, fixed in 4% PBS-buffered paraformaldehyde for 48 h and stored in 50% ethanol. For RNA isolation of bone tissue, humeri were flushed with PBS, immediately frozen with liquid nitrogen and stored at −80 °C until further analysis. All subsequent analyses (ELISA, RIA, microCT, bone biomechanics, histology/dynamic histomorphometry, RNA analysis of bone tissue) were performed in a blinded manner.

Animal procedures were approved by the institutional animal care committee of the Technische Universität Dresden and the Landesdirektion Sachsen (TVV 09/2021) and were performed according to the ARRIVE guidelines. We have complied with all relevant ethical regulations for animal use.

### Serum analysis

Serum concentrations of bone formation marker procollagen type 1 amino-terminal propeptide (P1NP) and bone resorption marker tartrate-resistant acid phosphatase (TRAP) were quantified using ELISAs according to the manufacturers´ protocols (IDS, Frankfurt/Main, Germany). Total T_4_ and total T_3_ serum concentrations were assessed using radioimmunoassays (IM1447 and IM1699 respectively; Beckman Coulter/DRG Instruments, Marburg, Germany) as previously described^3,44^. The lower limit of analysis was 10 nM and 0.22 nM for T_4_ and T_3_, respectively. Intra-assay coefficients of variations were below 7.5%.

### Analysis of femur length, bone mass and microarchitecture

The length of the femurs was measured postmortem using a caliper. Also, femurs and L4 were analyzed using micro-computed tomography (microCT) (vivaCT 40, Scanco Medical, Brüttisellen, Switzerland) with an X-ray energy of 70 kVp and isotropic voxel size of 10.5 µm (114 mA, 200 msec integration time). Following standard protocols from Scanco medical, trabecular (Tb.) and cortical (Ct.) bone parameters including bone volume/total volume (BV/TV), trabecular number (Tb.N), trabecular separation (Tb.Sp) and thickness (Tb.Th) were evaluated based on calculations including 100 scan slices. Trabecular bone analysis of L4 was conducted at the center contouring 50 slices above and 50 slices below the middle of the vertebral body. Femur trabecular bone parameters were assessed in the metaphyseal region starting 20 slices below the growth plate, while cortical bone was tested within the diaphyseal region midway between femoral head and distal condyles.

### Static and dynamic histomorphometry

After fixation in 4% PBS-buffered paraformaldehyde for 48 h, L4 vertebrae were decalcified in Osteosoft (Merck, Germany) for 7 days and dehydrated using ascending ethanol series. Subsequently, decalcified bones were embedded in paraffin and cut into 2 µm thick sections. Tartrate-resistant acid phosphatase (TRAP) staining was performed to quantify osteoclasts in an area of 0.90 mm² in the center of vertebrae using the Microscope Axio Imager M1 (Carl Zeiss Jena, Jena, Germany) and Osteomeasure software (OsteoMetrics, Atlanta, GA, USA). In addition, osteoblasts were identified by their morphology and localization along the bone surface and quantified in an area of 0.90 mm² in the center of vertebrae. Representative photos were taken using CellSens Entry Software Version 1.5 (OLYMPUS Cooperation, Shinjuku, Japan).

Dynamic bone histomorphometry was performed as described previously^26,43^. Five and two days before sacrifice, mice received intraperitoneal injections with the fluorochrome calcein (20 mg/kg BW; Sigma-Aldrich, Munich, Germany) that incorporates into newly formed bone. The third lumbar vertebrae were fixated in 4% PBS-buffered paraformaldehyde for 48 h and dehydrated via ascending ethanol series. Then, bones were embedded in methyl methacrylate (Technovit 9100, Heraeus Kulzer, Hanau, Germany) and cut into 7 µm sagittal sections for calcein label quantification of the trabecular bone. The mineralized surface/bone surface (MS/BS), the mineral apposition rate (MAR) and the bone formation rate/bone surface (BFR/BS) were quantified in an area of 1.44 mm² in the center of vertebrae according to established protocols^16,17,26,65,74^ using fluorescence microscopy (Microscope Axio Imager M1) and the Osteomeasure software. Representative photos were taken using Axio Vision 3.1 Software (Carl Zeiss Jena, Germany). Terminology and quantification procedures were conducted according to guidelines of the Nomenclature Committee of the ASBMR^75^.

### Bone biomechanics

Femurs were used for 3-point bend testing to determine cortical bone strength while L5 vertebrae were used for a compression test (Zwick Roell, Ulm, Germany). Beforehand, femurs and L5 vertebrae were rehydrated in PBS overnight. Femurs were then placed onto two supports with an intermediate distance of 6 mm and a mechanical force was applied vertically onto the middle of the femoral midshaft. Vertebrae were placed onto the center of the lower plate and pressure was applied via the upper platen. After reaching a preload of 1 N, the measurement started and continued with a load rate of 0.05 mm/s until failure. The maximal load (F_max_) was evaluated as an indicator of bone strength using testXpert II—V3.7 software (Zwick Roell, Ulm, Germany).

### Cell culture of primary murine osteoblasts and osteoclasts

Hind legs of Cre-negative and Cre-positive *Bmpr1a*^fl/fl^;Osx-Cre mice were collected and mesenchymal stromal cells (MSC) were obtained by flushing the bone marrow. To acquire primary murine osteoblasts, MSC were first cultured in growth medium (DMEM, 10% FCS, 1% Penicillin/Streptavidin (P/S)) until 80% confluence in a humidified atmosphere of 95% air and 5% CO_2_ at 37 °C. Subsequently, MSC were differentiated by adding an osteogenic cocktail (100 μM ascorbate phosphate, 5 mM β-glycerol phosphate, both from Sigma-Aldrich, Munich, Germany) to the growth medium over 7 days. For RNA and alkaline phosphatase (ALP) activity analysis, osteoblasts were then starved overnight in DMEM with 1% FCS and 1%P/S and treated the next day with 100 nM T_3_ (Sigma-Aldrich, Munich, Germany) over 48 h and 72 h, respectively. ALP activity was assessed as a marker of osteoblastic differentiation. After the 72 h treatment, cells were washed twice in PBS and then scraped in ALP lysis buffer (10 mM Tris-HCl pH 8.0, 1 mM MgCl_2_, and 0.5% Triton X-100) for isolation of total proteins. Subsequently, cell lysates were processed through a 24-gauge needle and centrifuged at 25,000 × *g* for 30 min at 4 ˚C. 10 µL of diluted supernatants (1:5 in distilled water) were incubated with 90 µl ALP substrate buffer (100 mM diethanolamine, 150 mM NaCl, 2 mM MgCl_2_, and 2.5 g/ml p-nitrophenylphosphate) for 30 min at 37 ˚C. Color change occurred due to hydrolysis of p-nitrophenylphosphate by enzymatic activity of ALP. Absorbance was measured at 410 nm using FluoStar Omega (BMG Labtech, Offenburg, Germany). Results were normalized to the total protein content that was quantified using Pierce™ BCA Protein Assay Kit (Thermo Fisher Scientific, Waltham, MA, USA) according to the manufacturer’s protocol.

To determine the mineralization capacity, MSC were differentiated and simultaneously treated with 100 nM T_3_ over 10 days, then fixed in 10% paraformaldehyde in PBS for 15 min and stained with 1% Alizarin Red S solution (pH 5.5, Sigma-Aldrich, Munich, Germany) for 30 min at room temperature. Repeated washing steps with distilled water were performed to remove excess stain. 100 mM cetylpyridinium chloride solution (Sigma-Aldrich, Munich, Germany) was used to dissolve incorporated calcium that was then quantified by photometric measurement at a wavelength of 540 nm using FluoStar Omega (BMG Labtech, Offenburg, Germany).

For osteoclast culture, long bones from *Bmpr1a*^fl/fl^;LysM-Cre mice were harvested and flushed with 1xPBS containing 5% FCS to isolate bone marrow. Following erythrocyte lysis with ACK lysis buffer (Thermo Fisher Scientific, Waltham, MA, USA), monocytes were isolated by magnetic cell separation (MACS) using Dynabeads^TM^ Biotin Binder and Anti-Mouse CD11b Clone M1/70 (both from Thermo Fisher Scientific, Waltham, MA, USA) according to the manufacturer’s protocols. Isolated monocytes were first cultured in a-MEM with stable glutamine, 10% FCS, 1% P/S and 25 ng/µl recombinant mouse macrophage colony-stimulating factor (M-CSF; R&D systems, Minneapolis, MN, USA). After 72 h, 50 ng/µl recombinant mouse RANKL (R&D systems, Minneapolis, MN, USA) was added to the medium to induce osteoclastogenesis. As soon as small osteoclast precursors were spotted (3−4 days later), cells were treated with 100 nM T_3_ over 48 h for RNA expression analysis.

### Indirect coculture experiments

To conduct indirect coculture experiments, supernatants were collected from over 10 days differentiated Cre-negative and Cre-positive *Bmpr1a*^fl/fl^;Osx-Cre osteoblasts that were cultured beforehand with or w/o 100 nM T_3_ over 48 h in a-MEM with 1% FCS and 1% P/S. To obtain osteoclasts as reported before^76^. bone marrow cells from long bones of 12-week-old male C57BL/6JRj were cultured in a-MEM supplemented with 10% FCS, 1% P/S, 2 mM L-glutamine and 10 ng/ml macrophage colony-stimulating factor (MCSF, from R&D Systems, Minneapolis, USA) over 24 h in a cell culture flask. Only the floating cells (mostly myeloid precursors) were collected and grown again in medium supplemented with 25 ng/ml M-CSF over 48 h. Osteoclastogenesis was induced by additionally adding 50 ng/ml receptor activator of nuclear factor kappa-B ligand (RANKL, from R&D Systems, Minneapolis, USA) over 4 days. Early osteoclasts were then treated with fresh a-MEM supplemented with 1% FCS, 1%P/S, 2 mM L-glutamine and osteoblast supernatants (1:1 ratio) supplemented with 25 ng/ml M-CSF and 50 ng/ml RANKL over 48 h and samples were used for subsequent RNA isolation and TRAP staining. As controls, we used d4 osteoclasts treated directly with 100 nM T_3_ (T3) over 48 h in a-MEM supplemented with 1% FCS, 1%P/S, 2 mM L-glutamine, 25 ng/ml M-CSF and 50 ng/ml RANKL.

TRAP-positive osteoclasts were quantified by TRAP staining at day 6 after starting RANKL treatment. At first, osteoclasts were fixated for 10 min in acetone/citrate buffer containing 37% paraformaldehyde at room temperature. Following two washing steps with tap water, TRAP staining was performed in the dark over 20 min using TRAP-Kit according to the manufacturer´s protocol (from Sigma-Aldrich, St. Louis, USA). TRAP-positive cells with three or more nuclei were counted as osteoclasts.

### RNA isolation, RT-PCR and quantitative real-time PCR

Total RNA from primary cells and bone tissue was extracted using ReliaPrep^TM^ RNA Tissue Miniprep System (Promega, Madison, WI, USA) and TRIzol reagent (Invitrogen, Darmstadt, Germany), respectively, following the manufacturer’s protocol and quantified using a Nanodrop spectrophotometer (Peqlab, Erlangen, Germany).

Five hundred nanograms of RNA were reverse transcribed using M-MLV Reverse Transcriptase (Promega, Madison, WI, USA) followed by GoTaq® qPCR Master Mix-based quantitative real-time PCR (Promega, Madison, WI, USA) according to established protocols (ABI7500 Fast, Applied Biosystems, Carlsbad, CA, USA). Primer sequences for mice are listed in supplemental Table S1. PCR conditions were: 50 °C for 5 min and 95 °C for 10 min followed by 40 cycles with 95 °C for 15 s and 60 °C for 1 min. Melting curves were evaluated using the following scheme: 95 °C for 15 s, 60 °C for 1 min and 95 °C for 30 s. Results were calculated based on the ∆∆CT method and are represented as x-fold increase normalized to β-actin and GAPDH mRNA levels.

### Statistical analysis and reproducibility

Data are presented as mean ± standard deviation. Statistical analysis comparing four groups are based on a Two-way ANOVA followed by Bonferroni’s multiple comparison post-hoc test using GraphPad Prism 9.0 (GraphPad, La Jolla, CA, USA). Values of *p* < 0.05 were considered statistically significant. Significant outliers were excluded based on Grubbs’ test provided in GraphPad by Dotmatics (https://www.graphpad.com/quickcalcs/grubbs1/).

Each in vitro experiment was performed at least twice with a number of >3 animals per group. All subsequent analyses (RNA analysis, ALP activity evaluation, Alizarin Red staining, TRAP staining) were conducted in a blinded fashion.

### Reporting summary

Further information on research design is available in the Nature Portfolio Reporting Summary linked to this article.

### Supplementary information

Supplementary Information

Description of Additional Supplementary Materials

Supplementary data

reporting summary

## Data Availability

All source data underlying the graphs and charts presented in the main figures can be found within the Supplementary Data. All other data are available from the corresponding author upon reasonable request.
