# Supplementary material for: Hyperthyroidism-driven bone loss depends on BMP receptor Bmpr1a expression in osteoblasts
Source: Commun Biol. 2024 May 8;7:548. doi: 10.1038/s42003-024-06227-0 (PMC11078941; doi:10.1038/s42003-024-06227-0)
Supplement: Supplementary file 1 — Supplementary Information [file 42003_2024_6227_MOESM1_ESM.pdf]

SUPPLEMENTAL FIGURES AND FIGURE LEGENDS

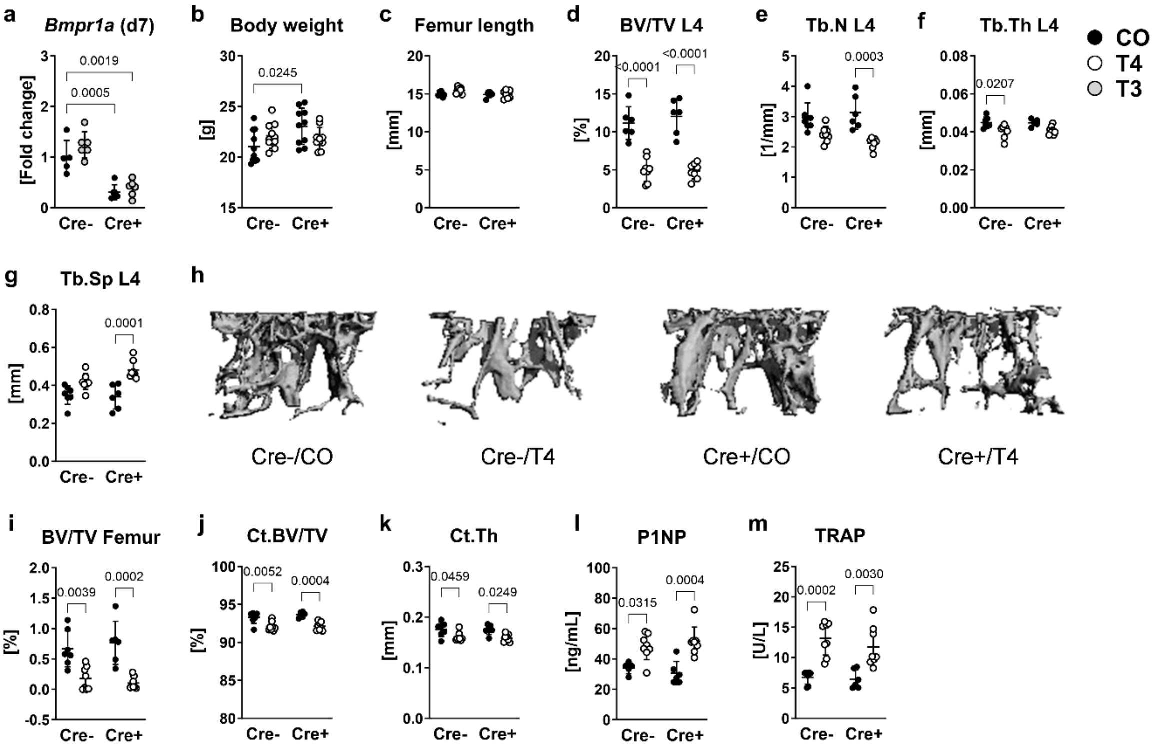

Figure S1. *Bmpr1a* deletion in osteoclast progenitors does not protect against bone loss in hyperthyroid female mice. (a) *In vitro*, knockout of *Bmpr1a* in primary osteoclasts derived from *Bmpr1a*$^{fl/fl}$;LysM-Cre mice at day 7 of differentiation, with or w/o treatment with 100 nM T_3 (T3) over 48 h, was verified using quantitative real-time PCR. To investigate possible sex-dependent effects, also 12-week-old female Cre-negative (Cre-) and Cre-positive (Cre+) *Bmpr1a*$^{fl/fl}$;LysM-Cre mice were rendered hyperthyroid (T4) by adding 1.2 µg/mL L-thyroxine into their drinking water over 4 weeks or received regular tap water and remained euthyroid (CO). (b) Body weight and (c) femur length were evaluated at the end of the experiment. Using microCT analysis, (d) bone volume per total volume (BV/TV), (e) trabecular number (Tb.N), (f) trabecular thickness (Tb.Th), and (g) trabecular separation (Tb.Sp) were determined at the fourth lumbar vertebra (L4). (h) Representative 3D reconstructions of the trabecular compartment of L4. Additionally, (i) trabecular BV/TV, (j) cortical bone volume over total volume (Ct.BV/TV) and (k) cortical thickness (Ct.Th) were measured at the femur. Serum concentrations of (l) bone formation marker P1NP and (m) bone resorption marker TRAP were analyzed by ELISA. Each dot indicates an individual mouse. *In vitro*: Cre-/CO: N=5; Cre-/T3: N=6; Cre+/CO: N=6; Cre+/T3: N=6. MicroCT & TRAP ELISA: Cre-/CO: N=7; Cre-/T4: N=8; Cre+/CO: N=6; Cre+/T4: N=8; P1NP ELISA: Cre-/CO: N=5; Cre-/T4: N=8; Cre+/CO: N=6; Cre+/T4: N=8. The horizontal lines represent the mean +/- SD. Statistical analysis was performed by Two-way ANOVA and selected p-values are shown within the graph.

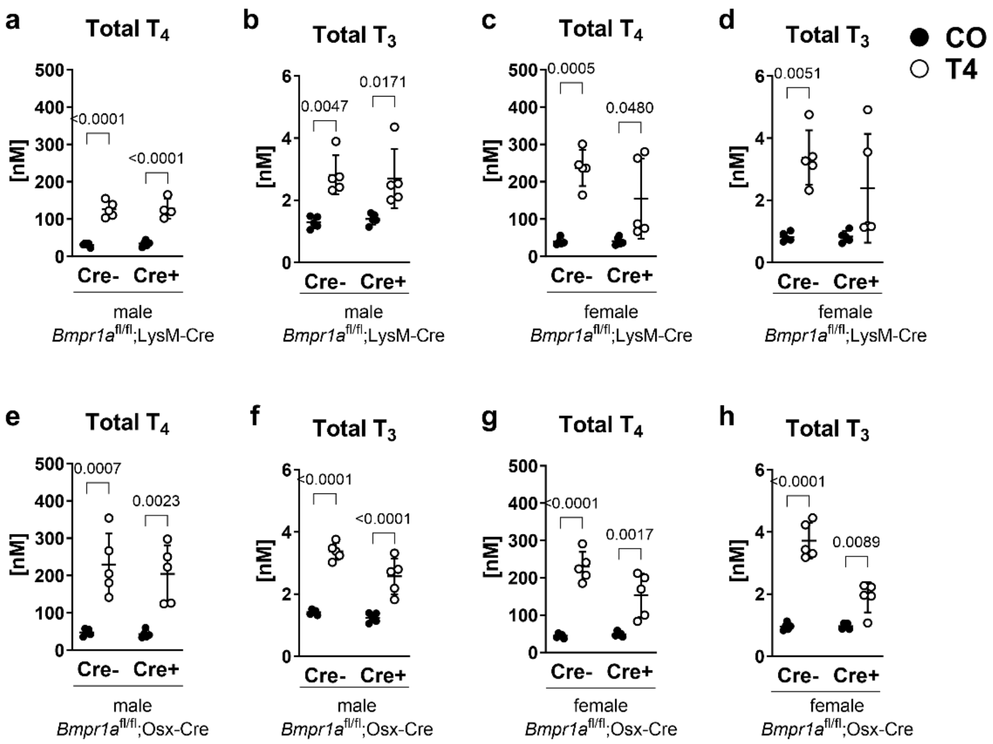

Figure S2. Serum thyroid hormone concentrations after L-thyroxine treatment in male and female *Bmpr1a*$^{fl/fl}$;LysM-Cre mice and *Bmpr1a*$^{fl/fl}$;Osx-Cre mice. Twelve-week-old male and female Cre-negative (Cre-) and Cre-positive (Cre+) *Bmpr1a*$^{fl/fl}$;LysM-Cre mice and *Bmpr1a*$^{fl/fl}$;Osx-Cre mice, respectively, were rendered hyperthyroid by adding 1.2 µg/mL L-thyroxine (T4) into their drinking water over 4 weeks or received regular tap water and remained euthyroid (CO). Serum concentrations of total T_4 and total T_3 from (a, b) male and (c, d) female *Bmpr1a*$^{fl/fl}$;LysM-Cre mice as well as (e, f) male and (g, h) female *Bmpr1a*$^{fl/fl}$;Osx-Cre mice were quantified at the end of the experiment using RIA. Each dot indicates an individual mouse. Cre-/CO: N=5; Cre-/T4: N=5; Cre+/CO: N=5; Cre+/T4: N=5. The horizontal lines represent the mean +/- SD. Statistical analysis was performed by Two-way ANOVA and selected p-values are shown within the graph.

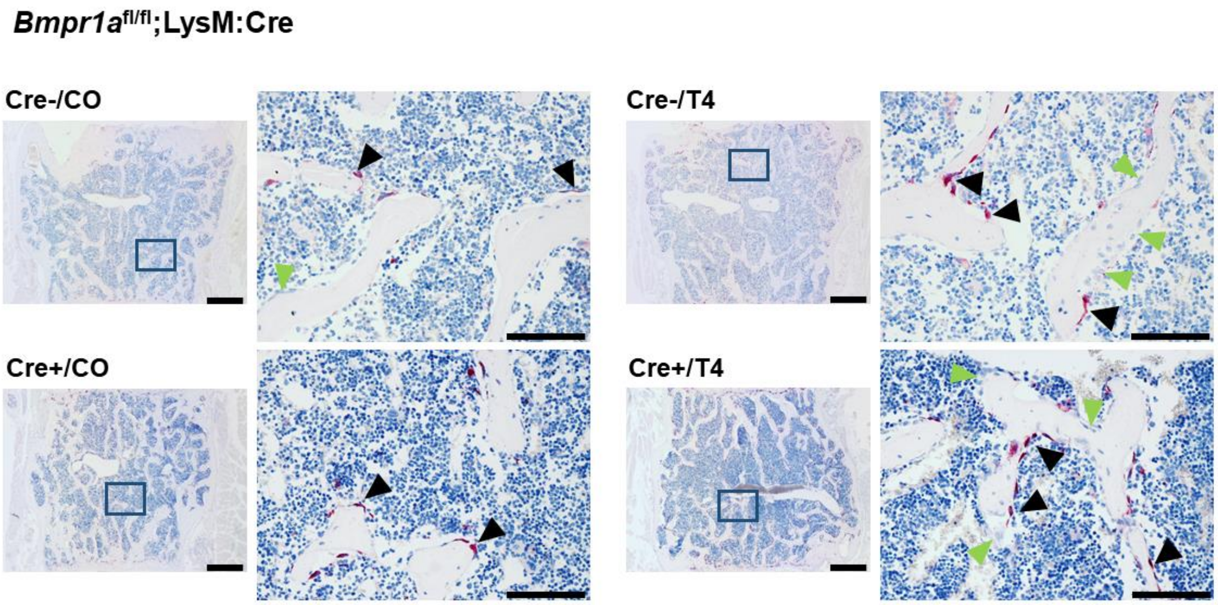

Figure S3. Representative images of TRAP-stained vertebral bone slides of male *Bmpr1a*$^{fl/fl}$;LysM-Cre mice. Twelve-week-old male Cre-negative (Cre-) and Cre-positive (Cre+) *Bmpr1a*$^{fl/fl}$;LysM-Cre mice were rendered hyperthyroid by adding 1.2 µg/mL L-thyroxine (T4) into their drinking water over 4 weeks or received regular tap water and remained euthyroid (CO). Tartrate-resistant acid phosphatase (TRAP) staining was performed to quantify osteoclasts (black arrows). Osteoblasts were identified by their morphology and localization along the bone surface (green arrows). Scale bar (overview images) = 500 µm. Scale bar (detailed images) = 100 µm.

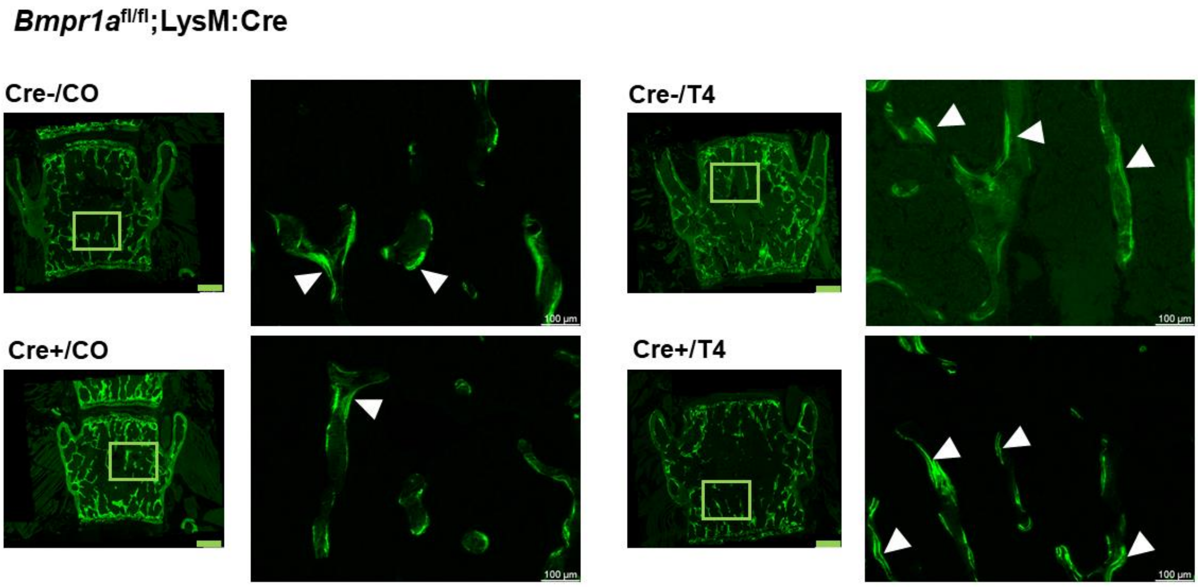

Figure S4. Representative images of Calcein-labeled vertebral bone slides of male *Bmpr1a*$^{fl/fl}$;LysM-Cre mice. Twelve-week-old male Cre-negative (Cre-) and Cre-positive (Cre+) *Bmpr1a*$^{fl/fl}$;LysM-Cre mice were rendered hyperthyroid by adding 1.2 µg/mL L-thyroxine (T4) into their drinking water over 4 weeks or received regular tap water and remained euthyroid (CO). Five and two days before sacrifice, mice received intraperitoneal injections with the fluorochrome calcein that incorporates into newly formed bone. Representative fluorescence images of calcein double labels are indicated by white arrows. Scale bar (overview images) = 500 µm. Scale bar (detailed images) = 100 µm.

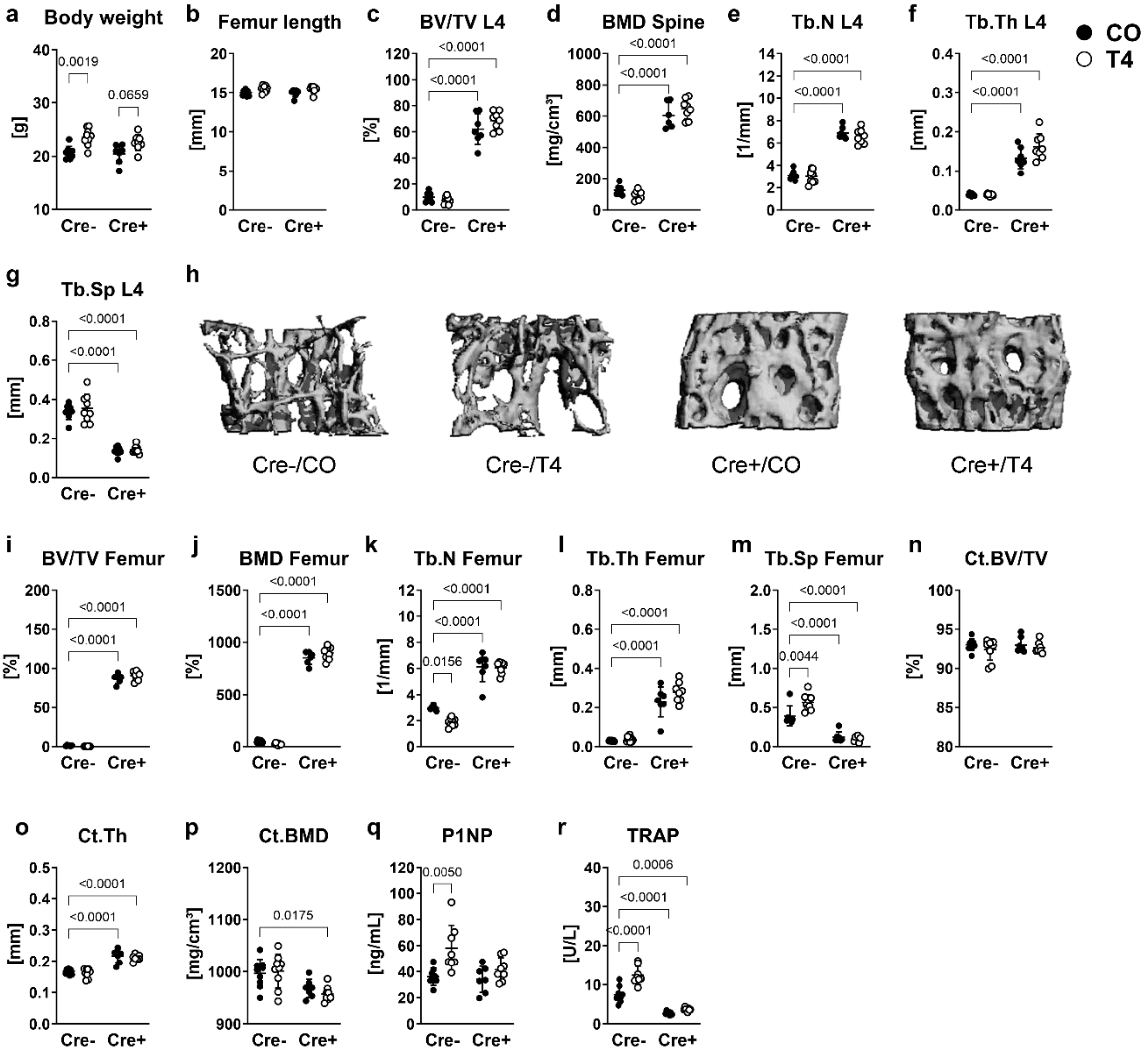

Figure S5. Conditional knockout of *Bmpr1a* in osteoprogenitors prevents osteoporosis in hyperthyroid female mice. Twelve-week old female Cre-negative (Cre-) and Cre-positive (Cre+) *Bmpr1a*$^{fl/fl}$;Osx-Cre mice were rendered hyperthyroid (T4) by adding 1.2 µg/mL L-thyroxine into their drinking water over 4 weeks or received regular tap water and remained euthyroid (CO). (a) Body weight and (b) femur length were measured at the end of the experiment. Using microCT analysis, (c) trabecular bone volume per total volume (BV/TV), (d) trabecular bone mineral density, (e) trabecular number (Tb.N), (f) trabecular thickness (Tb.Th), and (g) trabecular separation (Tb.Sp) were determined at the fourth lumbar vertebra (L4). (h) Representative 3D reconstructions of the trabecular compartment of L4. Furthermore, (i) trabecular BV/TV, (j) trabecular BMD, (k) Tb.N, (l) Tb.Th, (m) Tb.Sp, (n) cortical bone volume over total volume (Ct.BV/TV), (o) cortical thickness (Ct.Th) and (p) cortical bone mineral density (Ct.BMD) were measured at the femur. Serum concentrations of (q) bone formation marker P1NP and (r) bone resorption marker TRAP were assessed using ELISAs. Each dot indicates an individual mouse. MicroCT: Cre-/CO: N=9; Cre-/T4: N=9; Cre+/CO: N=7; Cre+/T4: N=8; ELISAs: Cre-/CO: N=8; Cre-/T4: N=8; Cre+/CO: N=6; Cre+/T4: N=8. The horizontal lines represent the mean +/- SD. Statistical analysis was performed by Two-way ANOVA and selected p-values are shown within the graph.

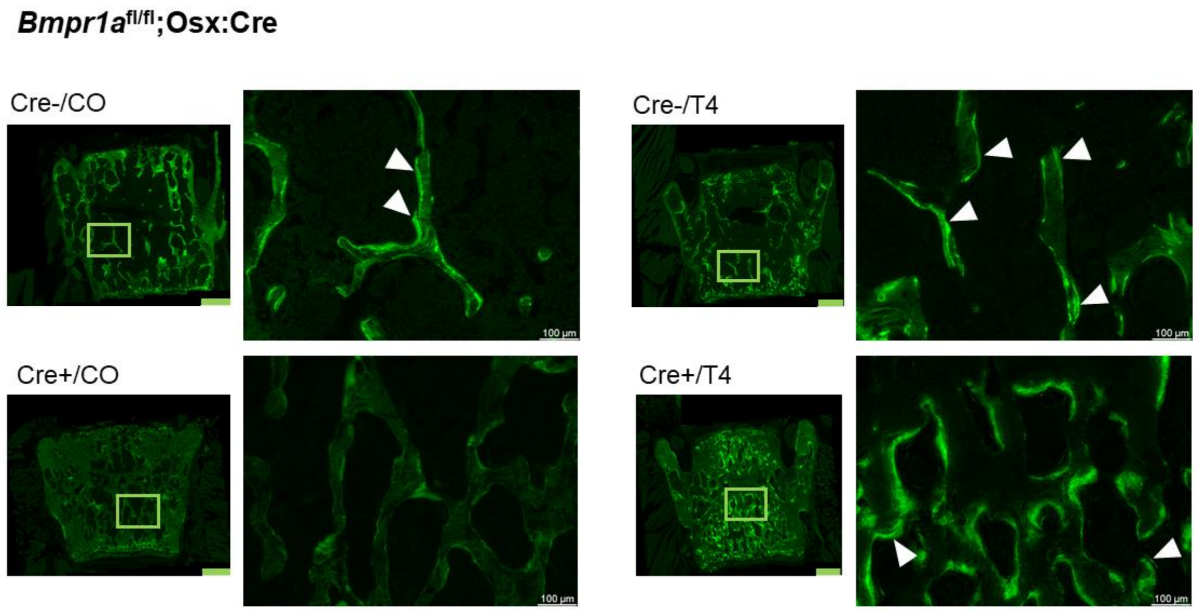

Figure S6. Representative images of Calcein-labeled vertebral bone slides of male *Bmpr1a*$^{fl/fl}$;Osx-Cre mice. Twelve-week-old male Cre-negative (Cre-) and Cre-positive (Cre+) *Bmpr1a*$^{fl/fl}$;Osx-Cre mice were rendered hyperthyroid by adding 1.2 µg/mL L-thyroxine (T4) into their drinking water over 4 weeks or received regular tap water and remained euthyroid (CO). Five and two days before sacrifice, mice received intraperitoneal injections with the fluorochrome calcein that incorporates into newly formed bone. Representative fluorescence images of calcein double labels are indicated by white arrows. Scale bar (overview images) = 500 µm. Scale bar (detailed images) = 100 µm.

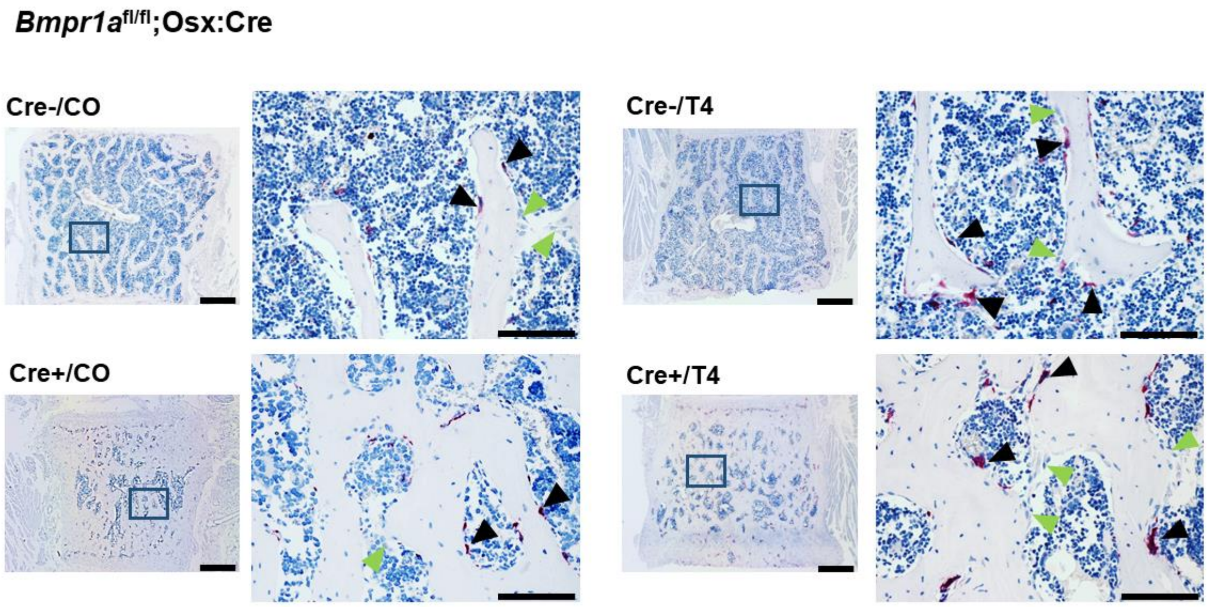

Figure S7. Representative images of TRAP-stained vertebral bone slides of male *Bmpr1a*$^{fl/fl}$;OsxCre mice. Twelve-week-old male Cre-negative (Cre-) and Cre-positive (Cre+) *Bmpr1a*$^{fl/fl}$;Osx-Cre mice were rendered hyperthyroid by adding 1.2 µg/mL L-thyroxine (T4) into their drinking water over 4 weeks or received regular tap water and remained euthyroid (CO). Tartrate-resistant acid phosphatase (TRAP) staining was performed to quantify osteoclasts (black arrows). Osteoblasts were identified by their morphology and localization along the bone surface (green arrows). Scale bar (overview images) = 500 µm. Scale bar (detailed images) = 100 µm.

SUPPLEMENTAL TABLE

Supplemental table S1. Primer sequences for mice used for quantitative real-time PCR.

Gene	RefSeq #	Sense	Antisense
Alpl	NM_007431	CTACTTGTGTGGCGTGAAGG	CTGGTGGCATCTCGTTATCC
Acp5	NM_001102405	ACTTGCGACCATTGTTAGCC	AGAGGGATCCATGAAGTTGC
beta-Actin	NM_007393	GATCTGGCACCACACCTTCT	GGGGTGTTGAAGGTCTCAAA
Bglab	NM_001032298	GCGCTCTGTCTCTCTGACCT	ACCTTATTGCCCTCCTGCTT
Bmpr1a	NM_009758	CTGGGAGCCTGTCTGTTCAT	TAGCATCATCTGGGCAGTGT
Ctsk	NM_007802	AAGTGGTTCAGAAGATGACGGGAC	TCTTCAGAGTCAATGCCTCCGTTC
Dcstamp	NM_029422	TCCTCCATGAACAAACAGTTCCAA	AGACGTGGTTTAGGAATGCAGCTC
Dio3	NM_172119	ATTTTGAGCGCCTCTACGTC	ATCATAGCGCTCCAACCAAG
Gapdh	NM_008084	AAGGTCATCCCAGAGCTGAA	CTGCTTCACCACCTTCTTGA
Id1	NM_010495	CCCACTGGACCGATCCGCCA	TGCTCTCGGTTCCCCAGGGG
Klf9	NM_010638	GGCTGTGGGAAAGTCTATGG	AAGGGCCGTTCACCTGTATG
Opg (Tnfrsf11b)	NM_008764	CCTTGCCCTGACCACTCTTA	ACACTGGGCTGCAATACACA
Sp7	NM_001348205	CTTCCCAATCCTATTTGCCGTTT	CGGCCAGGTTACTAACACCAATCT
Rankl (Tnfsf11)	NM_011613	CCAAGATCTCTAACATGACG	CACCATCAGCTGAAGATAGT
Runx2	NM_001271631	AAATGCCTCCGCTGTTATGAA	GCTCCGGCCCACAAATCT
Slc2a1	NM_011400	GCAGTTCGGCTATAACACTGG	AGAGACCAAAGCGTGGTGAG
Slc2a3	NM_011401	GGCATCGTTGTTGGGATTCT	TCTTGGACTCTCAGGGCAAA
Slc2a4	NM_009204	GGGTCCTTACGTCTTCCTTCT	CCTCTGGTTTCAGGCACTTT
