# Supplementary material for: Hyperthyroidism-driven bone loss depends on BMP receptor Bmpr1a expression in osteoblasts
Source: Commun Biol. 2024 May 8;7:548. doi: 10.1038/s42003-024-06227-0 (PMC11078941; doi:10.1038/s42003-024-06227-0)
Supplement: Supplementary file 2 — Description of Additional Supplementary Materials [file 42003_2024_6227_MOESM2_ESM.docx]

**Description of Additional Supplementary Files**

**File name:** Supplementary Data 1

**Description:** The source data behind the graphs in the main figures 1-5
